# Exogenous melatonin ameliorates steroid-induced osteonecrosis of the femoral head by modulating ferroptosis through GDF15-mediated signaling

**DOI:** 10.1186/s13287-023-03371-y

**Published:** 2023-07-03

**Authors:** Wenming Li, Wenhao Li, Wei Zhang, Hongzhi Wang, Lei Yu, Peng Yang, Yi Qin, Minfeng Gan, Xing Yang, Lixin Huang, Yuefeng Hao, Dechun Geng

**Affiliations:** 1 Department of Orthopedics, The First Affiliated Hospital of Soochow University, 188 Shizi Road, Suzhou, 215006 China; 2 Department of Orthopedics, Taizhou People’s Hospital, Taizhou, 225300 China; 3 Orthopedics and Sports Medicine Center, The Affiliated Suzhou Hospital of Nanjing Medical University, 242 Guangji Road, Suzhou, 215006 China

**Keywords:** Glucocorticoid, SONFH, Ferroptosis, Melatonin, MT2 receptor, GDF15

## Abstract

**Background:**

Ferroptosis is an iron-related form of programmed cell death. Accumulating evidence has identified the pathogenic role of ferroptosis in multiple orthopedic disorders. However, the relationship between ferroptosis and SONFH is still unclear. In addition, despite being a common disease in orthopedics, there is still no effective treatment for SONFH. Therefore, clarifying the pathogenic mechanism of SONFH and investigating pharmacologic inhibitors from approved clinical drugs for SONFH is an effective strategy for clinical translation. Melatonin (MT), an endocrine hormone that has become a popular dietary supplement because of its excellent antioxidation, was supplemented from an external source to treat glucocorticoid-induced damage in this study.

**Methods:**

Methylprednisolone, a commonly used glucocorticoid in the clinic, was selected to simulate glucocorticoid-induced injury in the current study. Ferroptosis was observed through the detection of ferroptosis-associated genes, lipid peroxidation and mitochondrial function. Bioinformatics analysis was performed to explore the mechanism of SONFH. In addition, a melatonin receptor antagonist and shGDF15 were applied to block the therapeutic effect of MT to further confirm the mechanism. Finally, cell experiments and the SONFH rat model were used to detect the therapeutic effects of MT.

**Results:**

MT alleviated bone loss in SONFH rats by maintaining BMSC activity through suppression of ferroptosis. The results are further verified by the melatonin MT2 receptor antagonist that can block the therapeutic effects of MT. In addition, bioinformatic analysis and subsequent experiments confirmed that growth differentiation factor 15 (GDF15), a stress response cytokine, was downregulated in the process of SONFH. On the contrary, MT treatment increased the expression of GDF15 in bone marrow mesenchymal stem cells. Lastly, rescue experiments performed with shGDF15 confirmed that GDF15 plays a key role in the therapeutic effects of melatonin.

**Conclusions:**

We proposed that MT attenuated SONFH by inhibiting ferroptosis through the regulation of GDF15, and supplementation with exogenous MT might be a promising method for the treatment of SONFH.

**Graphical Abstract:**

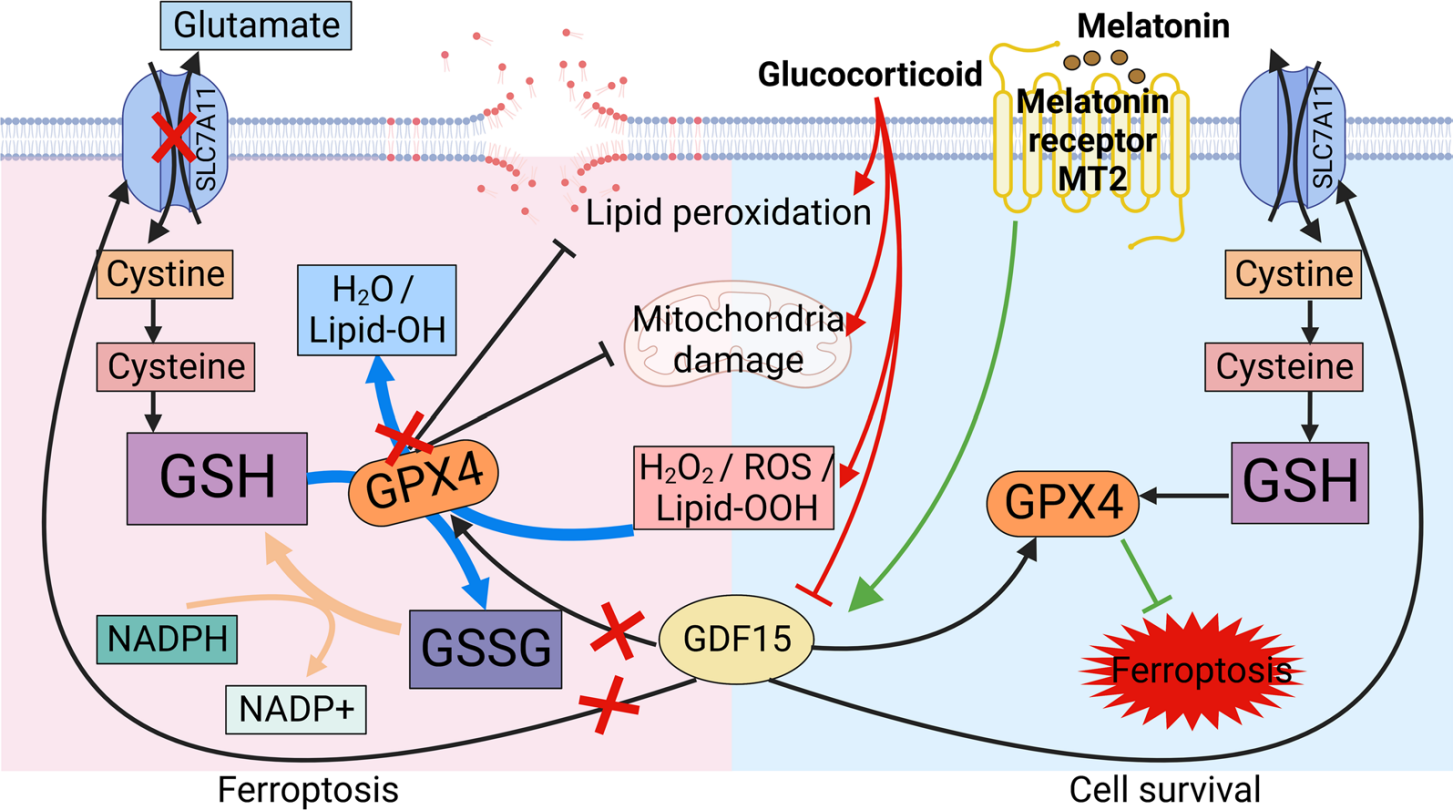

**Supplementary Information:**

The online version contains supplementary material available at 10.1186/s13287-023-03371-y.

## Introduction

Steroid-induced osteonecrosis of the femoral head (SONFH) is a refractory bone disease occurring globally, and is characterized by bone microstructure deterioration [1, 2]. Although it can be cured by total hip arthroplasty, patients currently tend to be younger and will live longer than artificial joints, which means that these young patients will undergo revision surgery one or more times. It is widely accepted that high-dose glucocorticoid (GC) treatment is the most common cause of SONFH [3–5]. GC has been extensively used in the treatment of autoimmune diseases such as systemic lupus erythematosus [6], owing to its anti-inflammatory and immunosuppressive properties. According to the existing studies, we can conclude that a high dose of GC can inhibit the osteogenesis of bone tissue, but the exact mechanism remains controversial [7, 8].

The balance between bone formation and resorption is crucial for performing skeletal function. Normal bone formation depends on osteoblast activity, but a high dose of GC can seriously suppress this process [8, 9]. There are three distinct stages of osteoblasts involved in the process of bone formation: osteoprogenitor, preosteoblast, and osteoblast. Finally, mature osteoblasts secrete extracellular matrixes and transform into osteocytes [10, 11]. Bone marrow mesenchymal stem cell (BMSC) are precursor cells of osteoblasts and play a crucial role in the supplementation of osteoblasts during bone formation and osteonecrosis repair [12]. Previous studies suggested that a high dose of GC inhibits BMSC proliferation, stimulates reactive oxygen species (ROS) production, and even leads to cell death [13, 14]. In addition, it has been reported that GC can also inhibit osteogenic gene transcription and translation [15, 16]. Recently, it was reported that ferroptosis, a type of programmed cell death different from apoptosis, can induce BMSC damage in multiple orthopedic diseases [17, 18]. Ferroptosis was first observed in a cell exposed to erastin, an inhibitor of voltage-dependent anion channel (VDAC), also known as a mitochondrial porin, and is characterized by iron-dependent cell death, during which mitochondrial destruction and lipid peroxidation are most significant [19, 20].

Ferroptosis is driven by the dysregulated redox state. Glutathione peroxidase 4 (GPX4), glutathione (GSH), and cystine/glutamate antiporter system (System Xc-) including solute carrier family 7 member 11 (SLC7A11), play an important role in cellular antioxidant system, which can rescue cells from ferroptosis [21, 22]. SLC7A11 transports cystine into the cytoplasm to promote antioxidant function of GPX4 that catalyze the reduction of hydrogen peroxide, organic hydroperoxides and lipid hydroperoxides, and thereby protect cells against ferroptosis [21, 22]. In addition, ACSL4 increases cell membrane polyunsaturated fatty acids content, and expands lipid peroxidation reaction to promote ferroptosis. Therefore, observation of changes in these proteins is necessary for the study of ferroptosis. Numerous studies claim that an uncontrolled redox state participates in the osteonecrosis process of SONFH, and treatment with antioxidants can effectively mitigate this process [23, 24]. In addition, increases in intracellular Fe^2+^ and ROS levels were observed in human umbilical vein endothelial cells (HUVECs) [25]. However, the relationship between BMSC damage and ferroptosis in SONFH is still unclear.

Furthermore, current pharmaceutical drugs have little effect on the treatment of SONFH, and most SONFH patients will have to undergo total hip arthroplasty (THA) surgery. Therefore, clarifying the mechanism and developing new drugs to prevent SONFH progression are urgently needed. Exploring therapeutic drugs for SONFH from approved drugs will help accelerate clinical translation. Melatonin (MT, N-acetyl-5-methoxytryptamine) is an indoleamine hormone that is mainly synthesized and secreted from the mammalian pineal gland during the night [26]. As studies look deeper into the function of MT, antioxidant, anti-inflammatory, and immunomodulatory properties were discovered, and MT has become a popular therapeutic drug and dietary supplement [27–29]. In addition, increasing studies have demonstrated that MT protects bone tissue against oxidative stress by antagonizing free radicals and stimulating cellular transcription factors to produce antioxidant enzymes [30–32]. MT mainly binds with its type 1 (MT1) and type 2 (MT2) melatonin receptors to achieve biological functions. The majority of research has indicated that MT2 may play more prominent regulatory roles in osteogenic action than MT1 [33, 34]. Therefore, the use of MT as a new strategy to prevent SONFH should be further explored.

In this study, we used methylprednisolone sodium succinate (MP) to establish a rat SONFH model and assessed whether MT could be a new candidate for SONFH treatment. Additionally, it is well known that GDF15 can be upregulated in response to injury as well as by metformin [35]. In recent years, GDF15 has received increased attention due to its anti-inflammatory, antioxidant and potential anti-obesity effects [35–37]. However, the association between GDF15 and SONFH pathogenesis has not been clarified. Overall, this study provides new insight into the pathogenesis of SONFH and a novel strategy for the prevention of SONFH as well as a new function of MT.

## Materials and methods

### Animal experiments

The study adhered to the ARRIVE guidelines and the ethical guidelines of the Laboratory Animal Center of Soochow University (Approved Number: 202209A0230, date: August 28, 2022). All animal experiments were carefully performed in all possible steps to avoid animal suffering. According to the guidelines for sample size calculations of Boston University, a total of 30 10-week-old male Sprague Dawley (SD) rats (weight: 350 ± 20 g) were used to establish the GC-induced ONFH model. We randomly divided the rats into the following three groups (n = 10/group): (1) Control, (2) MP, and (3) MT. Rats in the MP group were intraperitoneally injected with lipopolysaccharide (LPS, 40 µg/kg) (Sigma, St. Louis, MO, USA) from days 1 to 3 and received an intramuscular injection of MP (60 mg/kg/day) (Pfizer, USA) for the following 10 consecutive days. According to the methods of SONFH modeling, our SONFH modeling rate was stable at 80%. The animals in the MT group were treated with MT (50 mg/kg/day, i.p. injection) (Sigma) after the last MP injection. The dose of MT was based on a previous study [30]. The rats in the Control and MP groups received an injection of the same dose of saline. Six weeks after the establishment of the model, all rats were euthanized by intraperitoneal injection of 6% pentobarbital sodium (2.5–3.5 ml/kg), followed by the collection of their bilateral femoral heads for further studies.

### Micro-CT scanning

To evaluate the bone morphology of the femoral heads in rats, we performed high-resolution micro-CT (Alteslar, SKYSCAN 1176, Bruker, Konitch, Belgium) with the following scanning parameters: 18 µm, 70 kV, and 141 mA. The trabecular bone parameters, including bone mineral density (BMD, mg/cm^3^), bone volume (BV, mm3), bone volume per tissue volume (BV/TV, %), trabecular thickness (Tb. Th, mm), and trabecular number (Tb. N, 1/mm), were analyzed with CT Analyzer analysis software (CTAN, Bruker).

### Hematoxylin and eosin (H&E) and Masson staining

The femoral heads obtained from rats were fixed in 4% paraformaldehyde for 48 h and decalcified by using 10% diamine ethylene tetraacetic acid (EDTA, Sigma) for 4 weeks. Then, the femoral head samples were embedded in paraffin, sectioned into 7-µm-thick slices, and placed on slides. Finally, the femoral head slices were stained using H&E and Masson sealed with neutral resins. We observed the results under an AxioCam HRC microscope (Carl Zeiss, Oberkochen, Germany).

### Immunohistochemical (IHC) analyses

The processes of fixation, decalcification, embedding, and sectioning of the femoral heads were the same as those described for H&E staining. For immunohistochemical staining, the slices were subjected to deparaffinization; antigen retrieval; blocking in horse serum for 30 min; incubation with primary antibodies against Osterix (dilution 1: 100, Abcam: #ab209484), runt-related transcription factor 2 (RUNX2) (dilution 1: 100, Abcam: #ab236639), and osteocalcin (OCN) (dilution 1: 100, Abcam: #ab93876) for 12 h; and then incubation with the appropriate biotinylated secondary antibodies. All antibodies were purchased from Abcam (Cambridge, UK). Finally, the slices were stained with diaminobenzidine and counterstained using hematoxylin. Section images were acquired using an Axiovert 5 × and 20 × optical microscope (Zeiss, Germany), and the number of positive cells was used as a quantitative indicator.

### Cell extraction and culture

As previously described, BMSC was obtained from the femurs and tibias of 6-week-old healthy rats[38]. We cultured freshly extracted bone marrow cells using alpha minimum essential medium (α-MEM, HyClone, Logan, UT, USA) containing 10% fetal bovine serum and 1% penicillin/streptomycin for 24 h, and half of the medium was exchanged the next day. The remaining cells were cultured for another 3 days. After three days, the medium was completely exchanged, and the remaining adherent cells were the original BMSC. Then, the Petri dishes containing BMSC were placed in an incubator with a 5% CO_2_ atmosphere at 37 °C. The basic medium was replaced every 3 days, and BMSC passage was performed subsequently. Cells from passages 3–5 were used in this study.

### Cell cytotoxicity assay

Briefly, BMSC was cultivated in 96-well plates at a density of 5 × 10^3^ cells/well. The cells were then cultured with different concentrations of MP or MT for 24 h, 48 h, and 72 h. To detect BMSC activity, we added a solution containing 90% serum-free α-MEM and 10% CCK-8 solution to each well and then cultured the cells in the dark for 2 h at 37 °C. The absorbance value per well was measured using a microplate reader (BioTek, Winooski, VT, USA) at 450 nm.

### ROS detection assay

BMSC was cultured in 24-well plates (cell density: 1.5 × 10^4^ cells per well) for 48 h with different treatment in three replicate wells. The levels of intracellular ROS were evaluated using an ROS assay kit (2',7'-dichlorodihydrofluorescein diacetate, DCFH-DA) (Beyotime Biotech). The cells were incubated with medium containing 2,7-dichlorofluorescein diacetate (1:1,000 dilution) at 37 °C for 20 min in the dark after the medication interventions. After washing with phosphate-buffered saline (PBS) three times, we used an inverted fluorescence microscope to observe ROS-positive cells and a flow cytometer (Attune NxT, Thermo Fisher) to quantitatively analyze ROS levels.

Dihydroethidium was applied to detect the total ROS in the femoral head. After sacrifice, the femoral heads were quickly removed for embedding and freezing for frozen sectioning. Then, the femoral head was cut into 6 μm slices, dewaxed and rehydrated as described above. The rehydrated slices were stained with dihydroethidium at a concentration of 3 μM at 37 ℃ for 30 min. Finally, the sections were dehydrated and sealed, and fluorescence images were taken through confocal microscopy.

### Alkaline phosphatase (ALP) staining and ALP activity testing

BMSC was cultured in 24-well plates (cell density: 1.5 × 10^4^ cells per well) with different treatments in three replicate wells. After 10 days of culture using osteogenic induction medium (a-MEM supplemented with 10% fetal bovine serum, 50 μM ascorbic acid, 10 mm glycerol β-phosphate and 100 nm dexamethasone) containing different treatments, the BMSC was stained for ALP activity. The samples were fixed with 4% paraformaldehyde for 15 min. Then, after subjecting the samples to washing steps 2 to 3 times using PBS, all samples were immersed in 5-bromo-4-chloro-3-indolyl phosphate/nitro blue tetrazolium chloride working solution for 20 min in the dark. The images of ALP staining were analysed using an inverted microscope. ALP activity was detected by an alkaline phosphatase assay kit (Cell Biolab, San Diego, CA) at 450 nm optical density.

### Alizarin red S (ARS) staining

BMSC (2 × 10^4^ cells per well) was seeded in 24-well plates and stimulated with osteogenic induction medium containing different concentrations of MT and MP or other treatments for 18 days. After completion of induction, the samples were fixed in 4% paraformaldehyde for 20 min at 4 °C. Then, the cells were incubated in ARS staining solution (pH 4.2) for 20 min. Excess dye was removed by washing with PBS, and the samples were imaged with a light microscope. Cetylpyridinium chloride (10%) was applied to dissolve the ARS, and the solution was analyzed with a spectrophotometer at 562 nm.

### Western blot assay

BMSC was cultured in 6-well plates (cell density: 3 × 10^5^ cells per well), and proteins were extracted using radioimmunoprecipitation assay buffer (RIPA, NCM Biotech, Soochow, China). Then, proteins were obtained by collecting the supernatant after centrifugation (14,800 force) for 25 min at 4 °C. After determination and standardization of the total concentration of protein in each group, samples with equal protein contents were separated using 10% sodium dodecyl sulfate‒polyacrylamide gel electrophoresis (Beyotime Biotech, Haimen, China) and transferred onto nitrocellulose membranes. After blocking nonspecific binding sites with QuickBlock Blocking Buffer (Beyotime Biotech) for 1 h, the membranes were incubated with primary antibodies overnight at 4 °C. These primary antibodies were against GPX4 (dilution 1:1000, ABclonal: #A1933), ACSL4 (dilution 1:1000, ABclonal: #A20414), SLC7a11 (dilution 1:1000, ABclonal: #A2413), GDF15 (dilution 1:1000, ABclonal: # A0185) and β-actin (1:5000, Abcam: #ab8226). Then, the membranes were incubated with goat anti-rabbit IgG (H + L) HRP secondary antibodies (dilution 1:5000, MULTI SCIENCES: #70-GAR0072) for 1 h. Finally, we used an Enhanced ECL Chemiluminescent Substrate Kit (Yeasen, China: #36222ES76) to visualize the protein bands, and the relative gray values were measured using Image Lab 3.0 software (Bio-Rad, Hercules, CA, USA).

### Real-time quantitative PCR analysis

Total RNA was extracted using TRIzol (Invitrogen, USA), and then a NanoDrop-2000 (Thermo Fisher, USA) was used to measure the concentration of total RNA for reverse transcription. HiScript III RT SuperMix (Vazyme Biotech Co., China: #R323-01) was applied to synthesize cDNA, and PCR amplification was performed with Taq Pro Universal SYBR qPCR Master Mix (Vazyme Biotech Co., China: #Q712-02) in a CFX96 Touch Real-Time PCR Detection System (Bio-Rad, USA). Cycling systems were used as follows: 95.0 °C for 10 min at first, and then 40 cycles of 95.0 °C for 5 s, 60.0 °C for 30 s and 72 °C for 30 s. Primers were purchased from Sangon Biotech (Shanghai, China), and the sequences are presented in Table 1.

**Table 1:** Sequences of primers used for real-time qPCR analysis

Gene	Forward primer (5’‒3’)	Reverse primer (5’‒3’)
*Slc7a11*	TCCTTTCAAGGTGCCTCTG	TCCGAGTAAAGGGAGAGGA
*Acsl4*	AGTACAACTTTCCGCTTGTG	AAGCCTCAGACTCATTTAATCC
*Gpx4*	AGCAAGATCTGTGTAAATGGG	TTTGATGGCATTTCCCAGC
*Actb*	TCAGGTCATCACTATCGGCAAT	AAAGAAAGGGTGTAAAACGCA
*Sod2*	GAACCCAAAGGAGAGTTGC	CACAGCTGTCAGTTTCTCC
*Hmox1*	AGGAACACAAAGACCAGAG	CAGAGGTAGTATCTTGAACCAG
*Nox4*	TCTTCTGGTATACTCACAACCT	GATACTTCAACAAGCCACCC
*Runx2*	GAACCAAGAAGGCACAGAC	AATGCGCCCTAAATCACTG
*Sp7*	CTGCTTGAGGAAGAAGCTC	TATGGCTTCTTTGTGCCTC
*Alpl*	CTAGACACAAGCACTCCCA	CTAAGAGGTAGTCCACCCTG
*Bglap*	GAATAGACTCCGGCGCTACC	TCCTGGAAGCCAATGTGGTC

### Knockdown of GDF15

The shGdf15 lentivirus vector was purchased from Sangon Biotech (Shanghai, China), and the target sequence of shGdf15 as follows: GGCGTGTCACTGCAGACTTAT; reverse complement target sequence: ATAAGTCTGCAGTGACACGCC. Then, the cells were transfected with shGdf15 lentivirus according to the lentivirus infection protocols (www.addgene.org/protocols), and total RNA and proteins were harvested to validate knockdown efficiency.

### Cell immunofluorescence staining

BMSC was seeded in 24-well plates with 1.5 × 10^4^ cells per well. After treatment, BMSC was washed 3 times with PBS, immobilized with chilled 4% paraformaldehyde for 15 min and rinsed with PBS 2 times again. Then, the cells were blocked with immunofluorescence blocking solution for 1 h and cultured with primary antibodies against osteocalcin (OCN) (dilution 1:200, Abcam: #ab93876) or RUNX2 (dilution 1:200, Abcam: #ab192256) for 12 h at 4 °C. Finally, BMSC was incubated with a goat polyclonal secondary antibody against rat IgG (H&L) (Alexa Fluor® 488, Abcam) and TRITC-labeled phalloidin for 40 min at 37 °C, and nuclei were stained with DAPI (40,6-diamidino-2-phenylindole). Finally, the cells were photographed with a Zeiss laser scanning microscope (LSM510).

## Flow cytometry

BMSC was cultured in 6-well plates (cell density: 5 × 10^5^ cells per well) with different treatments. Cells were rinsed with ice-cold PBS and digested with trypsin for 1 min. Isolated cells were centrifuged (1800 rpm) and rinsed with ice-cold PBS two times. Then, the cells were cultured with different fluorescent reagents for subsequent flow cytometer analysis (Attune NxT, Thermo Fisher). FlowJo software version 10.6.2 (Tree Star, San Carlos, CA, USA) were utilized for data processing, and the results are presented as the mean fluorescence intensity (MFI).

Flow cytometry analysis of cell death: propidium iodide (PI) can pass through the cytomembrane of dead cells to bind with the nucleus, and the status of cell death was quantified by flow cytometry analysis. After cell collection, cells were stained with PI for 15 min according to the instruction manual. PI was purchased from the Annexin V-FITC/PI Apoptosis Detection Kit (Vazyme Biotech Co., Ltd., Nanjing, China).

Flow cytometry analysis of lipid peroxidation: To evaluate cell lipoperoxidation, 5 μM C11-BODIPY was incubated with cells in 6-well plates. One hour later, the cells were washed, digested and collected for flow cytometry analysis. C11-Bodipy can bind with cellular lipids, and once the cellular lipids are oxidized, C11-Bodipy emission fluorescence migrated from 590 to 510 nm. Therefore, the fluorescence intensity at 510 nm was positively associated with lipid peroxidation. C11 BODIPY 581/591 was purchased from ABclonal (Inc., China, #RM02821).

Flow cytometry analysis of mitochondrial membrane potential: The collected cells were resuspended in 10 µg/mL JC-1 for 15 min at 37 °C and 5% CO2. After incubation, the cells were rinsed with cell culture medium two times and analyzed by flow cytometry (emission: 590 nm). JC-1 was purchased from Yeasen Biotechnology (Shanghai, China, #36222ES76).

### Bioinformatic analysis

Datasets analyzed here were acquired from the Gene Expression Omnibus (GEO) databases (ncbi.nlm.nih.gov/geo/) by searching with the following keywords: 1. “(glucocorticoids AND osteoblast AND Homo sapiens) or (Osteonecrosis of the Femoral Head AND Homo sapiens)” for GC-induced changes in cell expression; 2. “(melatonin AND Homo sapiens)” for MT-induced changes in cell expression. Finally, through filtering with “Entry type: Series, and Study type: Expression profiling by high throughput sequencing”, GSE183359 and GSE190135 were selected for study on MT, and GSE112101 was chosen for study on GC-induced damage. GSE112101 includes information on 9 human cell types treated with MP (8500 μg/L, 22.7 μM) for 2 h and 6 h, and is based on the GPL11154 platform. GSE183359 and GSE190135 are RNA-seq of endothelial cells and hypertrophic scar fibroblasts treated with or without MT and based on the Illumina NovaSeq 6000.

Differentially expressed genes (DEGs) were analyzed with criteria of adjusted *P* < 0.05 and |logFC|≥ 2.0. Statistical analysis was carried out for each dataset. The Venn diagram was obtained from online painting tools (bioinformatics.psb.ugent.be/webtools/Venn/). Gene set enrichment analysis (GSEA) was performed by GSEA software to determine whether a set of ferroptosis-associated genes showed statistical significance between the GC-treated and normal groups. The ferroptosis-associated gene set was obtained from the FerrDb database (zhounan.org/ferrdb/current/).

### Statistical analysis

All results are expressed as the mean ± standard deviation (SD) and were analyzed using GraphPad Prism 9.0 software (GraphPad Software Inc. CA, USA). Homogeneity of variances tests and one-way analyses of variance (ANOVA) were used to measure the group variation, and Tukey’s multiple comparisons test was used to multiple comparison. A p < 0.05 was considered statistically significant.

## Results

### Methylprednisolone can destroy the ROS scavenging system and inhibit osteogenic differentiation in BMSC

**Fig. 1:**
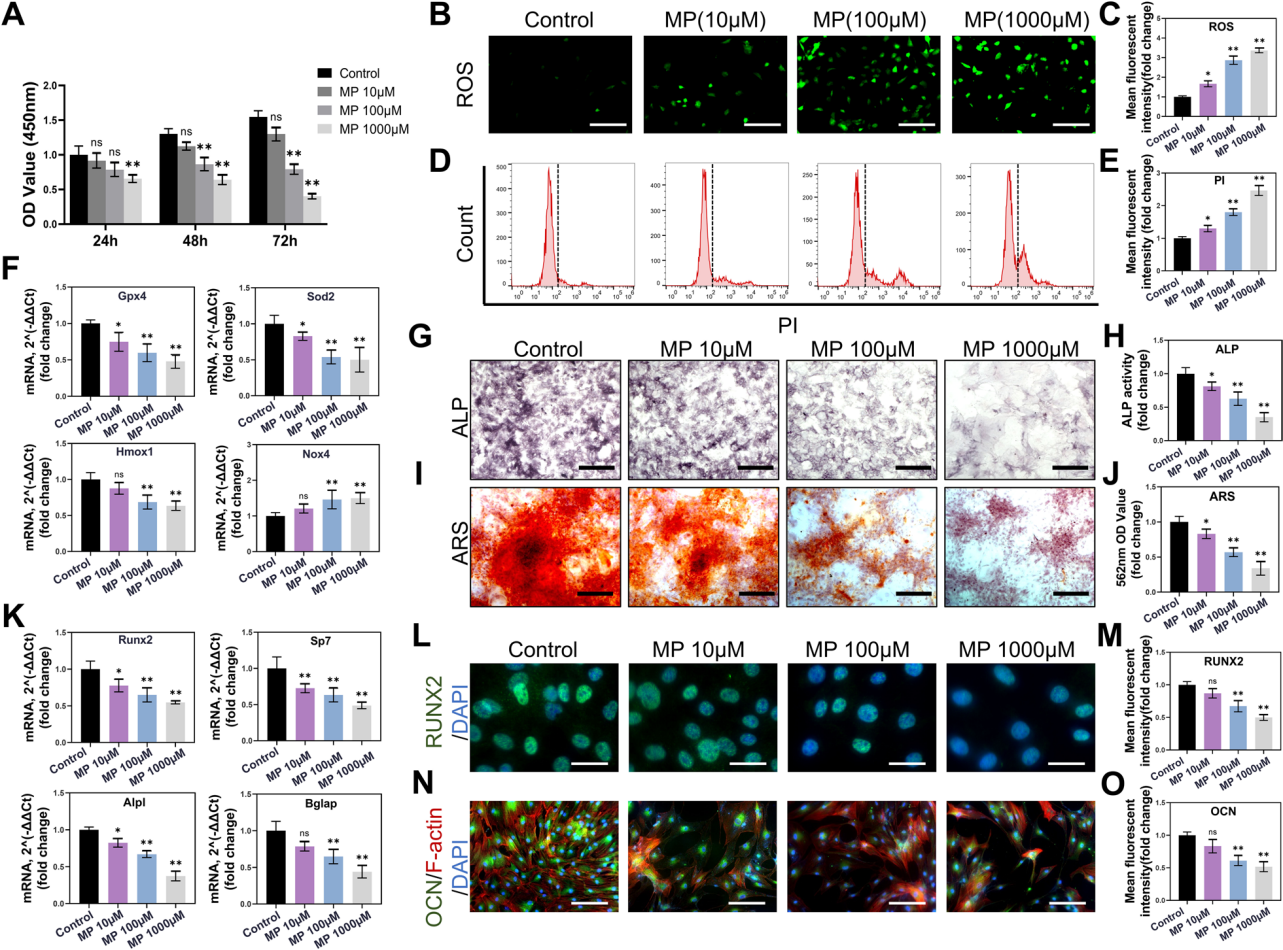
MP intensified oxidative stress and inhibited the osteogenesis of BMSC. **A** CCK-8 analysis was performed with various concentrations of MP in BMSC for 24 h, 48 h, and 72 h. **B** Immunofluorescence images of ROS. Intracellular ROS were stained with 2,7-dichlorofluorescein diacetate. (Scale bar: 100 μm) **C** Mean fluorescence intensity of ROS. **D** Flow cytometry analysis of PI staining. **E** Mean fluorescence intensity of PI. **F** qPCR analysis of Gpx4, Sod2, Hmox1 and Nox4. (β-actin was selected as an internal reference.) **G** ALP staining after 10 days of osteogenic induction. (Scale bar: 100 μm) **H** ALP activity. **I** ARS staining after 18 days of osteogenic induction. (Scale bar: 100 μm) **J** Quantitative analysis of ARS with spectrophotometer at 562 nm. **K** mRNA levels of Runx2, Sp7, Alpl and Bglap were detected after osteogenic induction. (β-actin was selected as an internal reference.) **L** Immunofluorescence staining of RUNX2 in BMSC. (Green: RUNX2, Blue: DAPI.) (Scale bar: 25 μm) **M** Mean fluorescence intensity of RUNX2. **N** Immunofluorescence staining of OCN in BMSC. **O** Mean fluorescence intensity of OCN. (Scale bar: 50 μm) (All bar graphs are the mean ± SD. *p < 0.05 and **p < 0.01 compared with the control group.)

Methylprednisolone (MP) is a commonly used GC in the clinic. In subsequent experiments, we applied MP to investigate the negative effects of GC on BMSC and SONFH. The CCK-8 results showed that 10 µM MP had no obvious effect on BMSC proliferation (*P* = 0.5599), and 100 µM MP significantly inhibited BMSC proliferation from 48 h (*P* < 0.01) (Fig. 1A).

To clarify the mechanism, we treated BMSC with MP in a concentration gradient. ROS detection assays showed that ROS levels in BMSC increased after treatment with MP in a concentration-dependent manner, especially at 100 µM and 1000 µM (*P* < 0.01) (Fig. 1B, C). At the same time, we observed that the cell death rate was positively correlated with ROS levels in BMSC (Fig. 1D, E). The delicate balance between ROS production and the ROS scavenging system is pivotal for maintaining normal physiological function. Proteins such as GPX4, heme oxygenase-1 (HO-1) and superoxide dismutase (SOD) have been proven to be important members of the antioxidant system [39, 40]. In the current study, the mRNA levels of Gpx4, Slc7a11 and Sod2 were significantly downregulated by MP treatment from 100 µM (*P* < 0.01) (Fig. 1F). In contrast, the levels of NADPH oxidase 4 (NOX4), a member of the NADPH oxidase family whose function is the production of ROS, were found to be upregulated from 100 µM (*P* < 0.01) (Fig. 1F). These results are consistent with previous studies and verify that the antioxidant capacity of BMSC is weakened after MP treatment in a concentration-dependent manner [24].

In addition, we further observed that the osteogenic ability of BMSC was also suppressed. ALP staining suggested that MP downregulated osteogenesis compared with the control, especially at high concentrations of MP (100 µM and 1000 µM) (*P* < 0.01) (Fig. 1G, H). In line with ALP staining, ARS staining showed that fewer calcium nodules formed in BMSC with osteogenic induction after treatment with 10 µM MP (*P* < 0.05), and especially at high concentrations of MP (100 µM and 1000 µM) (*P* < 0.01) (Fig. 1I, J). Since osteogenic markers are categorized into early-stage and late-stage markers, we selected the following genes to observe the different stages of osteogenic differentiation: Alpl, an early-stage marker of osteogenic differentiation; Bglap, a late-stage marker of osteogenic differentiation; Runx2 and Sp7, important transcription factors in the whole process of osteogenesis. In general, qPCR analysis for Runx2 and Sp7 at 3 days, for Alpl at 5 days, and for Bglap at 7 days. The result showed that all osteogenesis-related genes were significantly decreased from 100 µM MP treatment (*P* < 0.01) (Fig. 1K). Finally, we applied immunofluorescence to investigate changes in osteogenesis. The results showed that OCN and RUNX2 expression were negatively correlated with the concentration of MP intervention from 100 µM MP treatment (*P* < 0.01) (Fig. 1L–O).

### MT effectively alleviates MP-induced damage to BMSC in SONFH rats

**Fig. 2:**
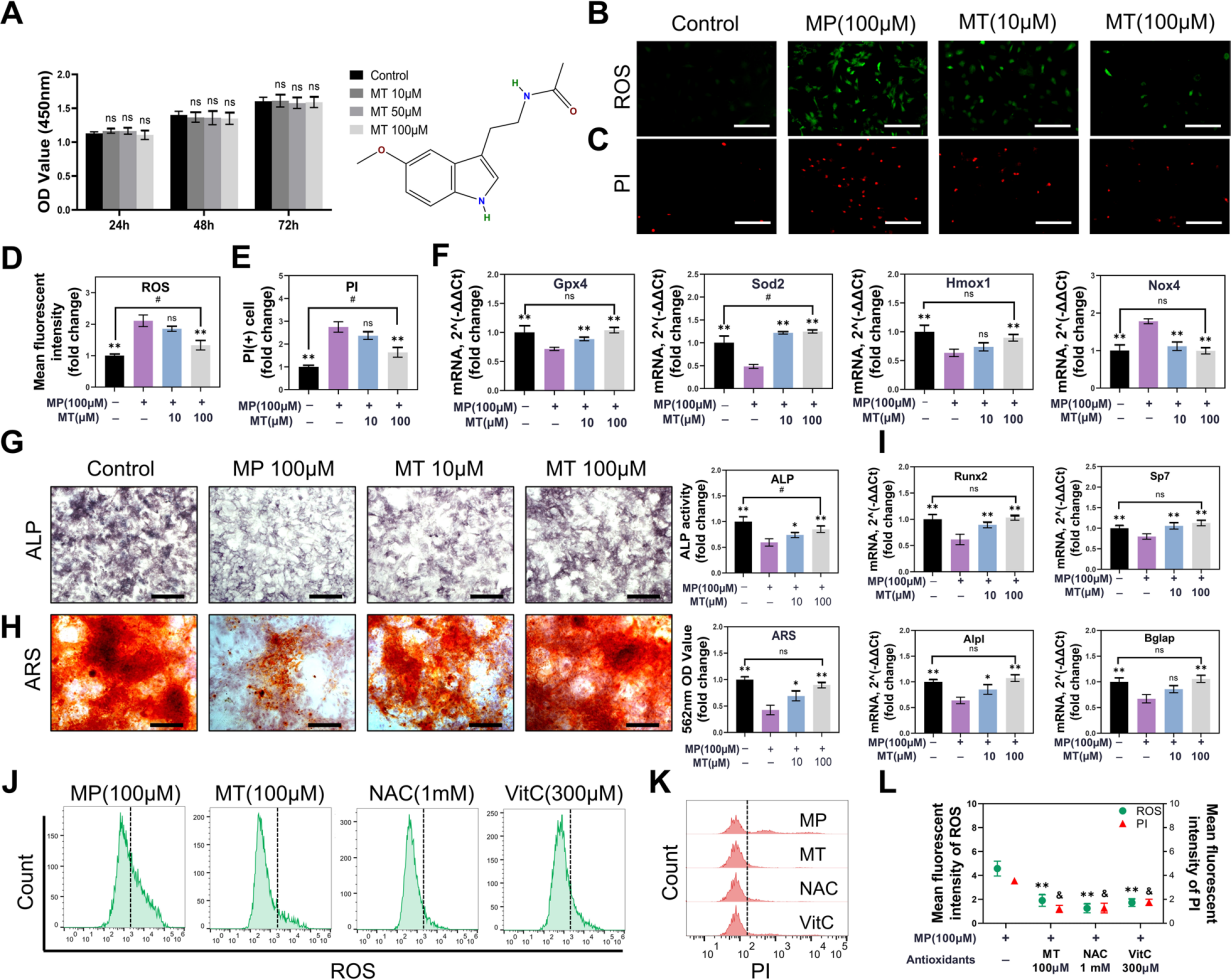
MT relieved the inhibitory effect of MP on BMSC. **A** The structural formula of melatonin and CCK-8 analysis were performed with various concentrations of MT in BMSC for 24 h, 48 h, and 72 h. **B** ROS detection with 100 µM MP and various concentrations of MT treatment. (Scale bar: 100 μm) **C** PI staining. (Scale bar: 100 μm) (D) Mean fluorescence intensity of ROS. **E** Number of PI (+) cells. **F** qPCR analysis of the Gpx4, Sod2, Hmox1 and Nox4 genes in BMSC. **G** ALP staining and quantitative analysis. (Scale bar: 100 μm) **H** ARS staining and quantitative analysis. (Scale bar: 100 μm) **I** qPCR analysis of Runx2, Sp7, Alpl and Bglap expression in BMSC. (β-actin was selected as an internal reference.) **J** Flow cytometry analysis of ROS staining. **K** Flow cytometry analysis of PI staining. **L** Mean fluorescence intensity of ROS and PI staining. (All bar graphs are the mean ± SD. *p < 0.05, **p and & *p* < 0.01 compared with the MP 100 µM group. ^#^*p* < 0.05 and ^##^*p* < 0.01 compared with the control group.)

MT is a neuroendocrine hormone secreted by the pineal gland and has become a popular dietary supplement because of a variety of health-promoting functions [27]. Accumulating evidence has verified the strong antioxidant capacity of MT [29, 41]. Therefore, we tried to apply the powerful antioxidant effect of MT to treat SONFH. In this study, MT in the range of 10–100 µM had no significant toxic effect on BMSC after 72 h of treatment, as shown by the CCK-8 assay (*P* = 0.5014) (Fig. 2A). Then, BMSC was subjected to MP at a concentration of 100 µM and supplemented with different concentrations of MT (10 µM and 100 µM) for 48 h. The results showed that 100 µM MT treatment could significantly reduce ROS production (*P* < 0.01) (Fig. 2B, D). Furthermore, PI staining showed that with MT treatment, MP-induced cell death was rescued in a concentration-dependent manner (Fig. 2C, E). At the same time, 100 µM MT returned the inhibitory state of ROS scavenging genes to normal, such as Gpx4, Slc7a11 and Sod2 (*P* < 0.01) (Fig. 2F). In contrast, the overexpression of Nox4 induced by MP was downregulated (*P* < 0.01) (Fig. 2F). These results indicate that MT effectively attenuated MP-induced oxidative stress by restoring the balance between the antioxidant system and ROS production. However, we found that 100 μM MT treatment could not reduce ROS levels to basal levels (*P* < 0.05) (Fig. 2D). Furthermore, PI staining results also indicated a significant difference in the 100 μM MT group compared to the control group (*P* < 0.05) (Fig. 2E). Therefore, although not playing a major role, other types of cell death might be involved in MP-induced BMSC damage.

In addition, increasing evidence suggests that MT is also involved in promoting osteogenic differentiation. To determine whether MT had this effect on MP-treated BMSC, we used several methods to detect osteogenesis indicators. ALP and ARS staining showed that the osteogenic potential of BMSC was markedly recovered after MT supplementation (*P* < 0.05) (Fig. 2G, H). Osteogenesis-related transcription factors, including Runx2 and Sp7, significantly upregulated in both 10 and 100 µM MT groups (*P* < 0.01), while Bglap and Alpl increased in 10 µM MT and significantly upregulated in 100 µM MT group (*P* < 0.01) compared with the MP group (Fig. 2I). Collectively, MT not only alleviates oxidative damage in BMSC but also promotes osteogenesis-related genes to relieve the inhibitory effect of MP on osteogenesis. To observe antioxidant capacity in a more intuitive way, we selected two other admitted antioxidants, N-acetyl-L-cysteine (NAC) and vitamin C (VitC, ascorbic acid), to compare with MT group. The flow cytometry results showed that MT, NAC and VitC all possess excellent properties in eliminating ROS (*P* < 0.01) (Fig. 2J, L). In cell death detection, the three antioxidants all effectively rescued the BMSC from MP damage compared with the MP group (*P* < 0.01), and MT seemed to have a better protective effect, although there was no significant difference from the NAC (*P* = 0.682) and VitC groups (*P* = 0.082) (Fig. 2K, L).

**Fig. 3:**
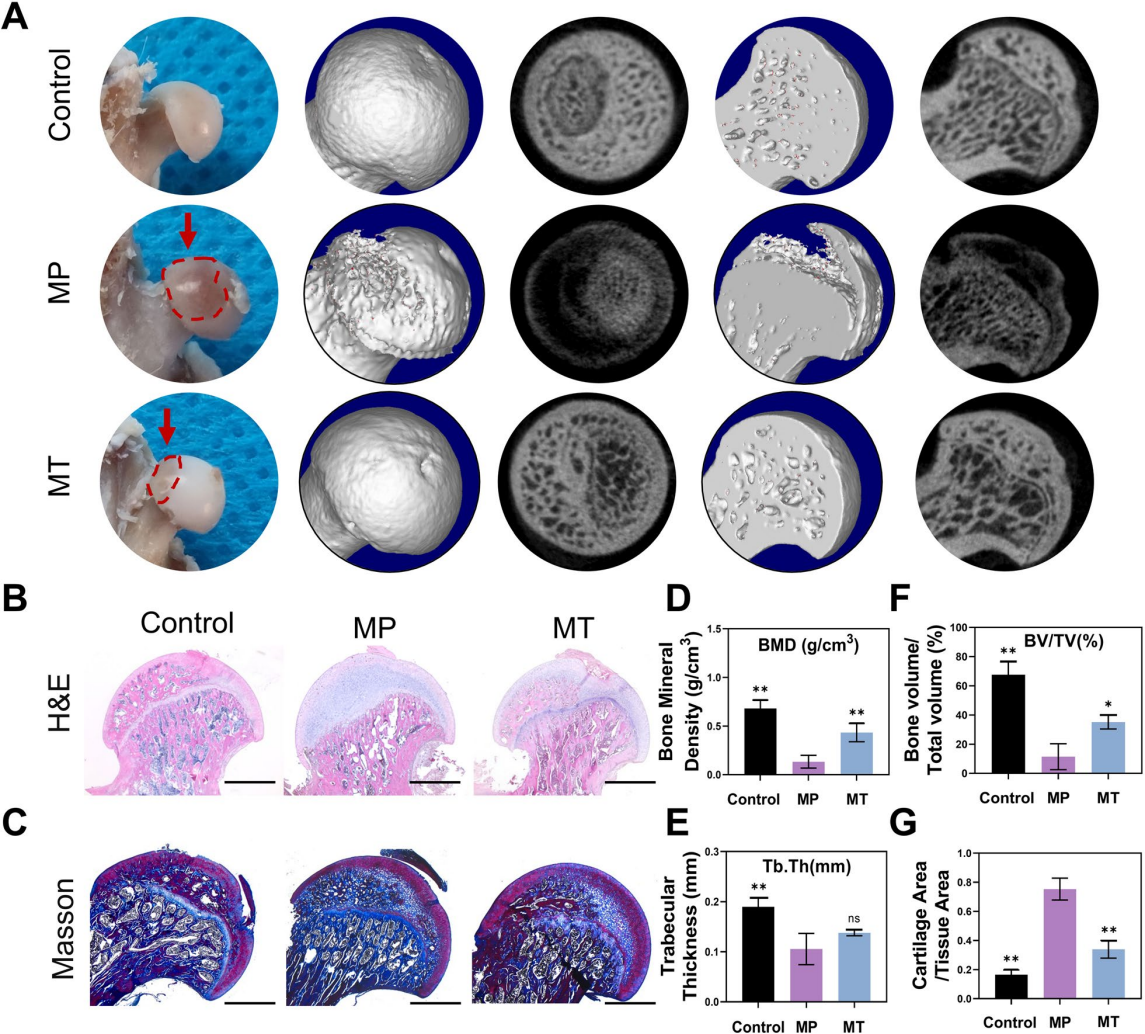
MT alleviated SONFH in rats. **A** Image of femoral heads and micro-CT 3D reconstruction. **B** H&E staining. **C** Masson staining. **D** Bone mineral density (BMD, mg/cm3). **E** Trabecular thickness (Tb. Th, mm). **F** Bone volume/tissue volume (BV/TV, %). **G** Cartilage area/tissue area. (Scale bar: 800 μm. *p < 0.05 and **p < 0.01 compared with the MP group.)

Animal experiments further validated the protective effect of MT. The SONFH model was established and confirmed with micro-CT, H&E and Masson staining (Fig. 3A–C). Trabecular changes in the subchondral area of the femoral heads were measured for subsequent analysis. No osteonecrosis was observed in the control group, but it was obvious in the MP group. In addition, parameters associated with bone quality, including BMD (Fig. 3D), Tb. Th (Fig. 3E) and BV/TV (Fig. 3F) were markedly decreased in the MP group (*P* < 0.01). In contrast, MT treatment alleviated osteonecrosis and increased BMD (*P* < 0.01), BV/TV (*P* < 0.05), and Tb. Th (*P* = 0.0549) (Fig. 3D–F). Consistent with the micro-CT results, histomorphological analysis with H&E and Masson staining showed that MT significantly reduced the cartilage area and increased the trabecula area in the femoral head (*P* < 0.01) (Fig. 3B, C, G).

**Fig. 4:**
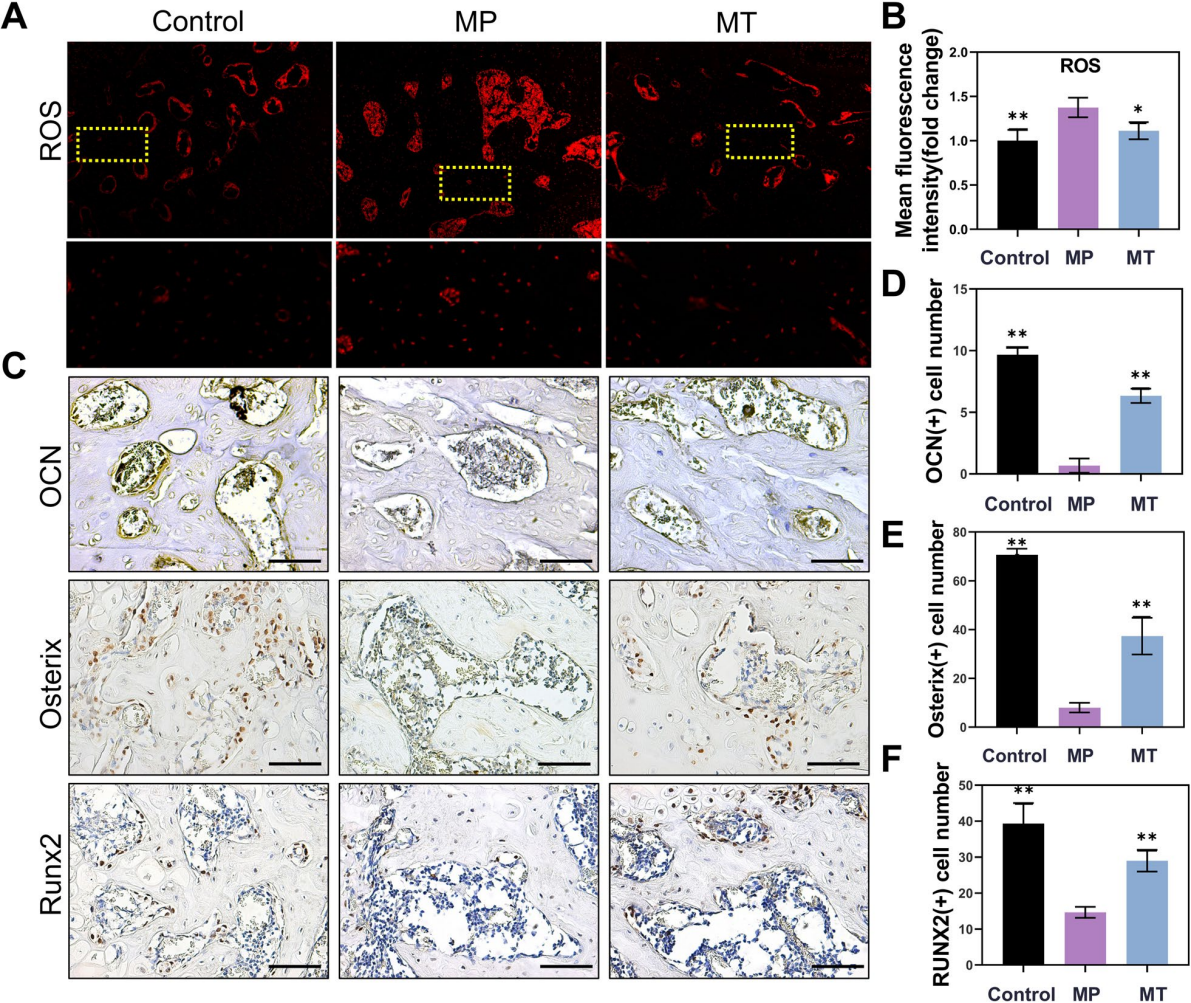
MT weakened ROS production and promoted osteogenesis in SONFH rats. **A** The ROS level of the femoral head was detected by DHE. **B** Representative immunohistochemical staining for OCN, Osterix and RUNX2 in decalcified bone sections. **C** Mean fluorescence intensity of ROS. **D** OCN-positive cells, **E** Osterix-positive cells and **F** RUNX2-positive cells. (*p < 0.05 and **p < 0.01 compared with the MP group. Scale bar: 100 μm.)

In addition, the ROS level in the femoral head was also significantly upregulated after MP treatment, and this change was alleviated in the MT group (*P* < 0.05) (Fig. 4A, B). Similarly, immunohistochemical staining of osteogenic differentiation-related proteins, such as RUNX2, Osterix, and OCN, was decreased in the MP group, whereas the expression of these proteins was significantly increased after MT treatment (*P* < 0.01) (Fig. 4C–F).

These results suggest that MT can be a promising candidate for preventing femoral head necrosis against GC injury. In summary, MT was effective in reducing MP-induced ONFH in rats, and this effect may be achieved by enhancing antioxidant capacity and promoting osteogenic differentiation.

### Ferroptosis participated in the pathogenesis of SONFH, and MT could suppress ferroptosis

**Fig. 5:**
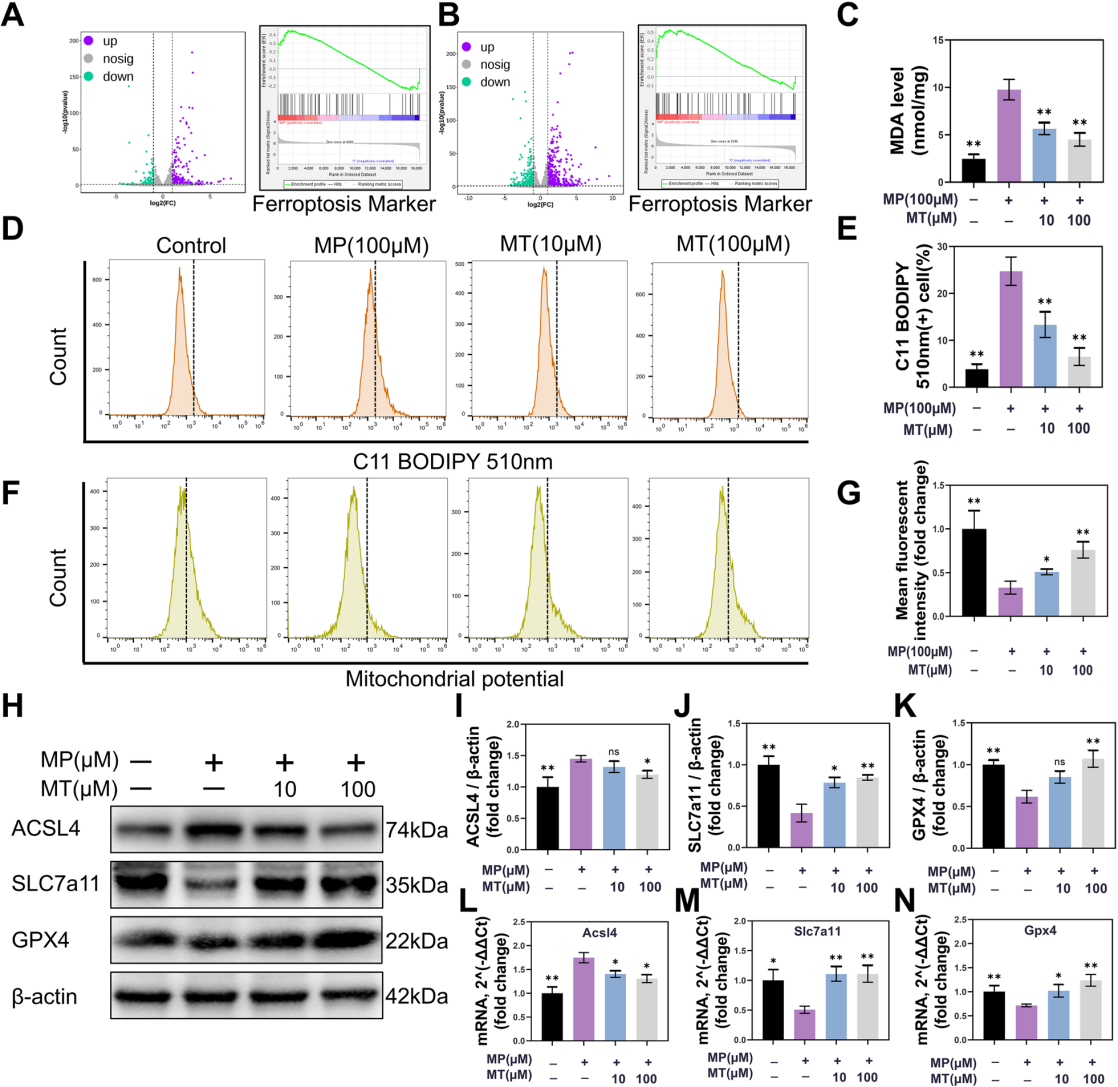
MT worked against MP-induced ferroptosis in BMSC. **A**, **B** DEGs and GSEA analysis. GSE112101 is RNA-seq data including nine cell populations from humans after in vitro exposure to MP (8500 μg/L, 22.7 μM) for two time points, 2 h and 6 h. Here, we selected 2 h and 6 h of osteoblast RNA-seq to conduct DEGs and GSEA analysis. (**|**log(2)(fold change)**|**≥ 1, *p* value ≤ 0.05). **C** MDA detection. **D** Flow cytometry analysis of C11-BODIPY staining. (Excitation: 500 nm, emission: 510 nm) **E** Quantitative analysis of C11-BODIPY oxidation. **F** Flow cytometry analysis of mitochondrial potential through JC-1. (Excitation: 585 nm, emission: 590 nm) **G** Quantitative analysis of JC-1. **H** After treatment with 100 µM MP and 10 µM MT or 100 µM MT for 72 h, BMSC was collected for western blot analysis. **I**–**K** The fold change in the relative gray levels of ACSL4, SLC7a11 and GPX4 compared to the no intervention group. (β-actin was selected as an internal reference.) **L**–**N**. qPCR analysis of Acsl4, Slc7a11 and Gpx4 expression in BMSC. (β-actin was selected as an internal reference.) (*p < 0.05 and **p < 0.01 compared with the 100 μM MP treatment group. Full-length blots are presented in Additional file 2: Figures S3–S6.)

To clarify the mechanism of ROS overproduction, bioinformatics methods were performed to analyze MP-treated RNA-seq. After strictly searching and screening the Gene Expression Omnibus (GEO) database, GSE112101 RNA-seq profiles were selected. GSE112101 is an expression profile obtained from high-throughput sequencing of 9 cell populations exposed to MP (8500 μg/L, 22.7 μM) for 2 h or 6 h. With the analysis of osteoblast RNA-seq from GSE112101, a total of 341 differentially expressed genes (DEGs) were found in osteoblasts exposed to MP for 2 h, and 811 DEGs were found in osteoblasts exposed to MP for 6 h (Fig. 5A, B). Then, gene set enrichment analysis (GSEA) illustrated that ferroptosis-associated genes (obtained from FerrDb, which is a ferroptosis database) were more positively related to the MP-treated group than to the control in both the 2 h and 6 h groups (Fig. 5A, B).

Ferroptosis is characterized by mitochondrial destruction and uncontrolled lipid peroxidation. Therefore, malondialdehyde (MDA) and C11-BODIPY 581/591, oxidation-sensitive fluorescent fatty acid analogs, were used to detect lipid peroxidation, and JC-1 was applied for mitochondrial potential detection. Both the MDA and C11-BODIPY results indicated that the state of lipid peroxidation was significantly increased after 100 μM MP treatment (*P* < 0.01), and MT attenuated this state in a concentration-dependent manner (*P* < 0.01) (Fig. 5C-E). Mitochondrial potential detection with JC-1 illustrated that MP treatment decreased the mitochondrial potential (*P* < 0.01), indicating that mitochondria were destroyed (Fig. 5F). When the BMSC was treated with MT, the mitochondrial potential was recovered (*P* < 0.05) (Fig. 5F, G). Moreover, the western blot results showed that ACSL4, SLC7A11 and GPX4 were obviously changed in BMSC after culture with a high dose of MP (*P* < 0.01) (Fig. 5H–K; full-length blots are presented in Additional file 2: S3–S6). Previous studies confirmed that ACSL4, SLC7A11 and GPX4 play important roles in regulating lipid peroxidation [42]. Fortunately, MT treatment could obviously alleviate the decrease of SLC7A11 and GPX4 at 100 μM (*P* < 0.01), and relieve the increase of ACSL4 at 100 μM (Fig. 5H–K). The qPCR results also showed that MT restored the changes in Acsl4, Slc7a11 and Gpx4 expression after culture with a high dose of MP (*P* < 0.05) (Fig. 5L–N). These results verified that ferroptosis was involved in MP-induced BMSC damage and that MT could alleviate this damage by regulating antioxidant proteins.

**Fig. 6:**
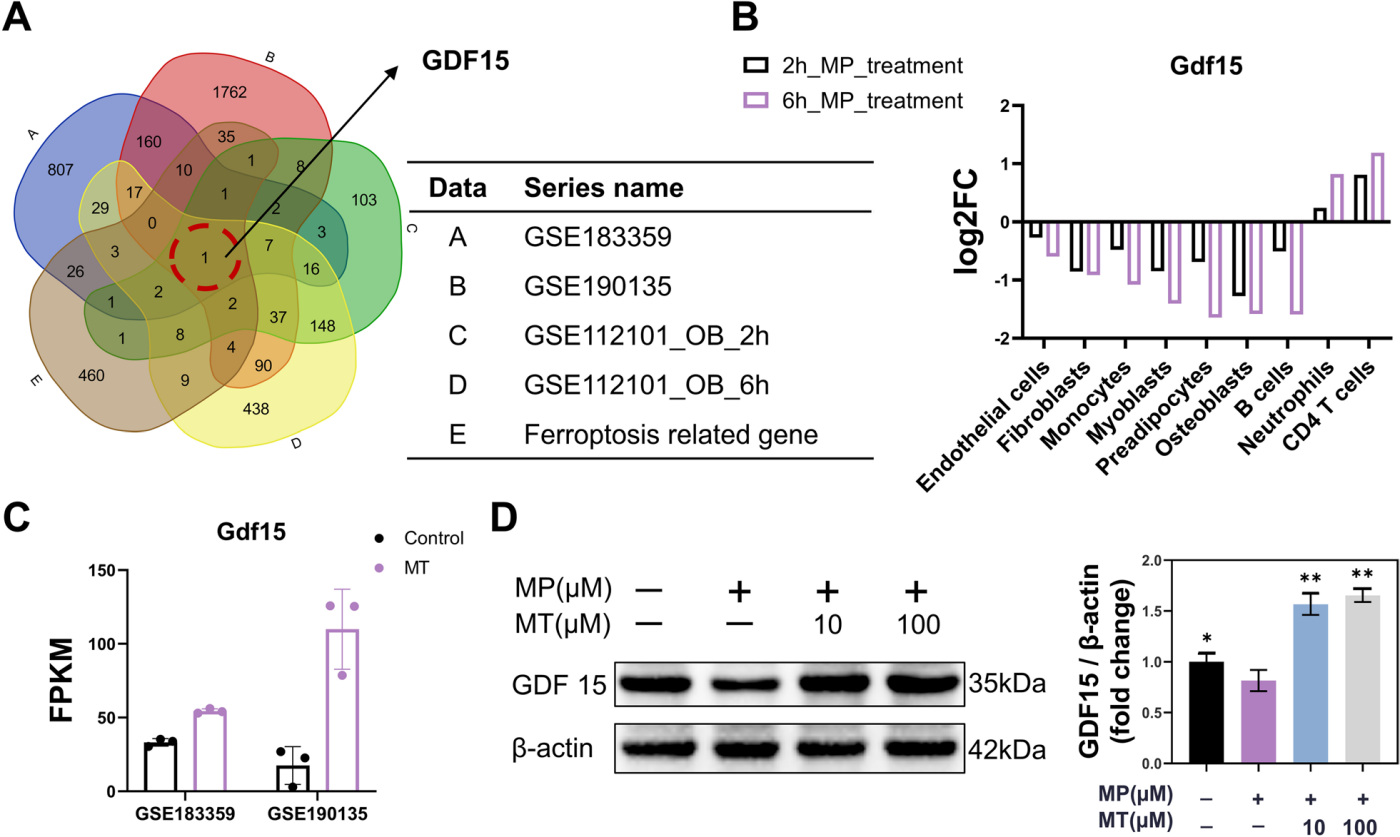
MT suppressed MP-induced ferroptosis by increasing GDF15 expression. **A** Venn diagram: Gdf15 is the only gene from the intersection result. **B** Changes in the expression of the Gdf15 gene in 9 human cell types after high-dose MP treatment, including osteoblasts, endothelial cells, fibroblasts, myoblasts, monocytes, neutrophils, preadipocytes, and CD4 T and B cells. (The gene expression profiles of these cells were obtained from GSE112101.) **C** Changes in the expression of the Gdf15 gene after melatonin treatment. (The gene expression profiles were obtained from GSE183359 and GSE190135.) **D** Protein expression of GDF15 in BMSC treated with 100 µM MP and 10 µM MT or 100 µM MT for 48 h. (β-actin was selected as an internal reference. *p < 0.05 and **p < 0.01 compared with the 100 μM MP treatment group. Full-length blots are presented in Additional file 2: Figures S7, S8.)

To clarify the key genes in MT treatment, MT-treated RNA-seq datasets were searched and analysed. Through rigorous screens, GSE183359 and GSE190135 were screened and analyzed for DEGs with selection criteria (q value < 0.05 and |log(2)(fold change)|≥ 1). A total of 1039 and 2137 DEGs were found in GSE183359 and GSE190135, respectively. Subsequently, Venn analysis was conducted to calculate the intersections among ferroptosis datasets (FerrDb), GSE112101_OB_2h (osteoblasts treated with MP for 2 h), GSE112101_OB_6h (osteoblasts treated with MP for 6 h), GSE183359 and GSE190135 (Fig. 6A). Finally, the GDF15 gene was screened from these five groups.

GDF15 belongs to the TGFβ family, whose function has recently gained attention due to its anti-inflammatory, antioxidant and potential anti-obesity effects [35]. Therefore, we hypothesized that GDF15 might play an important role in the process by which high-dose GC affect cell function. We found that GDF15 was significantly decreased in osteoblasts, endothelial cells, fibroblasts, myoblasts, monocytes, preadipocytes and B cells (Fig. 6B). The gene expression profiles of these cells were obtained from GSE112101, in which RNA-seq of these cells was collected at two time points after treatment with MP or control for 2 h and 6 h. In contrast to GC treatment, intervention with MT significantly increased GDF15 expression in GSE183359 and GSE190135 (Fig. 6C). These results led us to speculate that GDF15 is downregulated in the process of GC-induced ferroptosis and that MT treatment might increase the expression of GDF15, which exerts the ability to neutralize ROS and alleviate ferroptosis. Western blot analysis demonstrated that GDF15 expression decreased in MP-treated BMSC (*P* < 0.05), and MT treatment significantly increased the expression of GDF15 in both 10 and 100 µM MT treatment (*P* < 0.01) (Fig. 6D; full-length blots are presented in Additional file 2: Figures S7, S8). In summary, MT protected BMSC from ferroptosis induced by MP through the regulation of GDF15.

### Melatonin MT2 receptor participates in the regulation of ferroptosis and osteogenic differentiation through GDF15 signaling

MT is mainly secreted from the pineal gland and works mainly by interacting with two high-affinity G protein-coupled receptors, the melatonin MT 1 and MT 2 receptors. Previous studies have shown that in the osteogenesis process, MT mainly plays its role through MT2 receptors. To further clarify whether MT alleviates the MP-induced oxidative stress and inhibition of osteogenic differentiation through MT2, we blocked MT2 with the receptor inhibitor luzindole (10 µM).

**Fig. 7:**
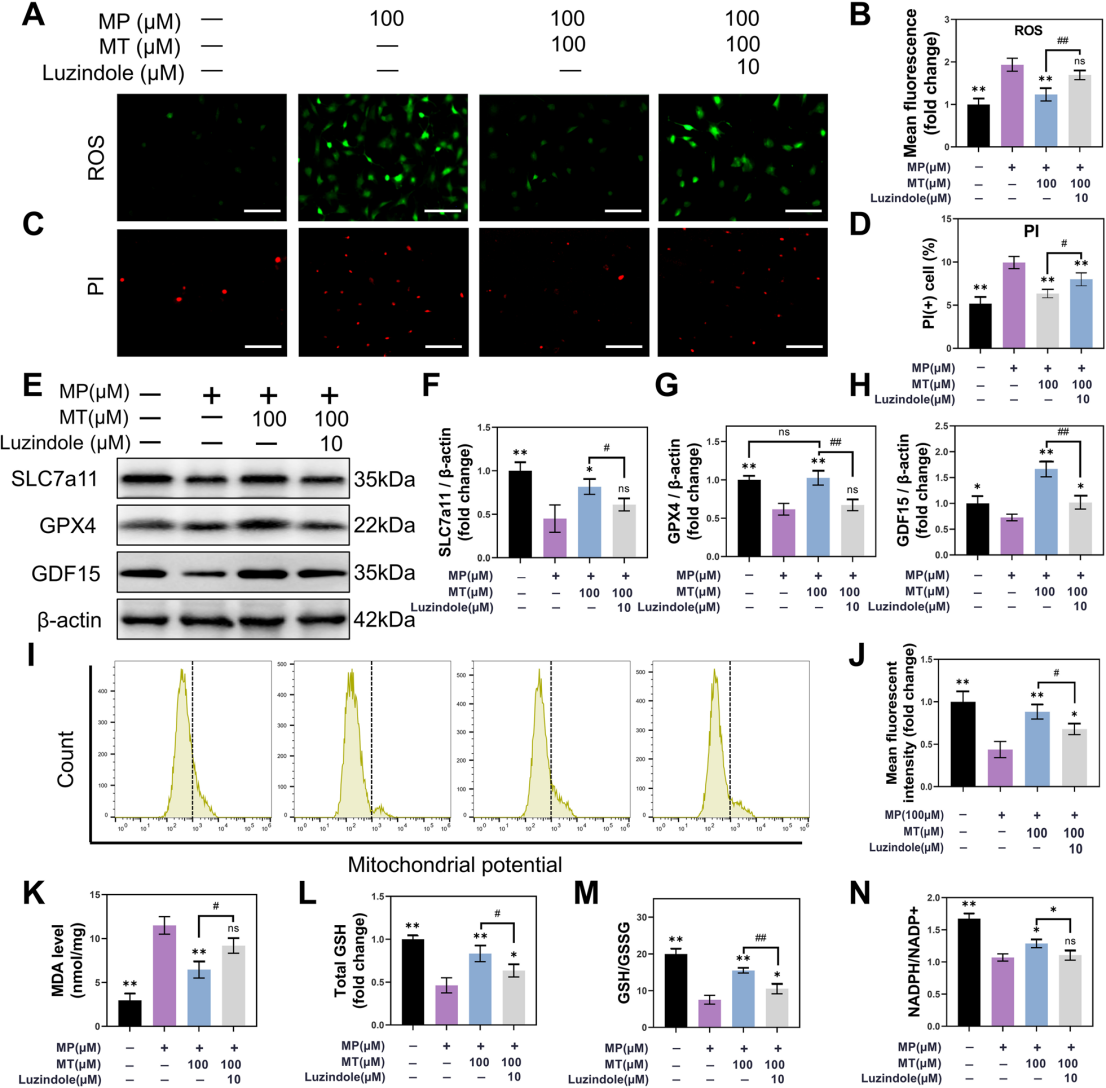
MT alleviated MP-induced ferroptosis through the melatonin MT2 receptor. **A**, **B** ROS detection. (Scale bar: 100 μm) **C**, **D** PI staining and analysis. (Scale bar: 100 μm) **E**–**H** Western blot analysis of SLC7a11, GPX4 and GDF15 in BMSC after treatment with or without 10 μM luzindole, 100 μM MP and 100 μM MT. (β-actin was selected as an internal reference.) **I** Flow cytometry analysis of mitochondrial potential by JC-1. (Excitation: 585 nm, emission: 590 nm) **J** Quantitative analysis of JC-1. **K** Detection of MDA. **L** Detection of total GSH. **M** Detection of the GSH/GSSG ratio. **N** Detection of the NADPH/NADP + ratio. (*p < 0.05 and **p < 0.01 compared with the 100 μM MP treatment group. ^#^*p* < 0.05 and ^##^*p* < 0.01 compared with the 100 μM MT treatment group. Full-length blots are presented in Additional file 2: Figures S9–S11.)

The ROS staining result showed that ROS levels were upregulated in MP intervention, and were alleviated by MT treatment. Luzindole intervention once again raised the ROS levels, despite MT treatment (*P* < 0.01) (Fig. 7A, B). The PI staining results were also consistent with the ROS staining results that inhibition of MT2 receptors diminished the therapeutic effect of MT (*P* < 0.05) (Fig. 7C, D). Furthermore, SLC7A11 and GPX4 protein levels were upregulated with MT supplementation but downregulated after MT2 was blocked with luzindole (*P* < 0.05) (Fig. 7E–G; full-length blots are presented in Additional file 2: Figures S9–S11). Meanwhile, GDF15 expression was also blocked by luzindole (*P* < 0.01) (Fig. 7E, H). Therefore, we speculated that MT could upregulate GDF15 expression by MT2 receptor to promote SLC7A11/GSH/GPX4 axis to mitigate ferroptosis. Mitochondrial potential increased in MT treatment, but decreased after luzindole intervention (*P* < 0.05) (Fig. 7I, J). MDA detection results also indicated that the inhibitory effect of MT on lipid peroxidation was blocked by the luzindole (*P* < 0.05) (Fig. 7K). GSH testing further showed that MT treatment increased the total GSH levels, which enhanced the ability of BMSC to maintain redox homeostasis (*P* < 0.01) (Fig. 7L). Then, luzindole intervention blocked the effects of MT on GSH (*P* < 0.05) (Fig. 7L). Finally, GSH/GSSG and NADPH/NADP + ratios were detected. High-dose MP reduced the GSH/GSSG and NADPH/NADP + ratios, MT treatment restored GSH/GSSG (*P* < 0.01) and NADPH/NADP + (*P* < 0.05) levels, and luzindole intervention reduced GSH/GSSG (*P* < 0.01) and NADPH/NADP + (*P* < 0.05) ratios again (Fig. 7M, N). In summary, luzindole could block the effect of MT on attenuating oxidative stress and ferroptosis, which further suggested that MT2 plays an important role in MT-mediated antioxidant stress.

**Fig. 8:**
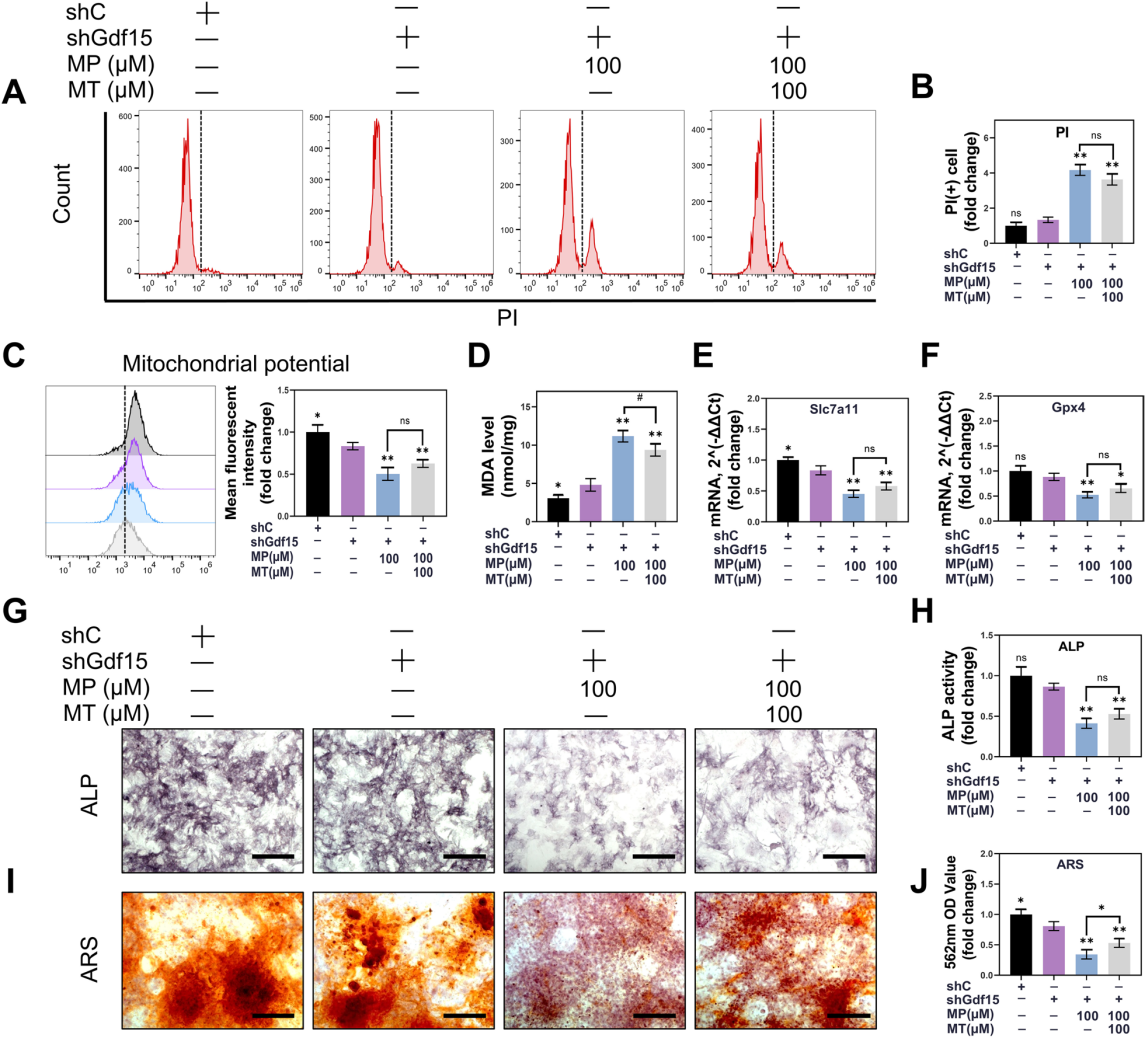
MT alleviated MP-induced ferroptosis through the GDF15 signaling. **A** Flow cytometric analysis of PI staining. **B** Quantitative analysis of PI (+) cells. **C** Flow cytometry analysis of mitochondrial potential by JC-1 and quantitative analysis. (Excitation: 585 nm, emission: 590 nm) **D** MDA detection. **E**, **F** qPCR analysis of Slc7a11 and Gpx4 expression in BMSC. (β-actin was selected as an internal reference.) **G** ALP staining. (Scale bar: 100 μm) **H** Quantitative analysis of ALP activity. **I** ARS staining. (Scale bar: 100 μm) **J** Quantitative analysis of ARS. (*p < 0.05 and **p < 0.01 compared with the 100 μM MP treatment only group. ^#^*p* < 0.05 and ^##^*p* < 0.01 are comparisons between 100 μM MP and 100 μM MT treatment groups after Gdf15 knockdown.)

As shown in Additional file 1: Figure S1A, B, OCN expression in BMSC was reduced, after MT2 receptor was blockade by luzindole (*P* < 0.01). Compared with the increased osteogenesis after MT supplementation, ALP activity and the mineralized nodules of ARS staining were also downregulated when MT2 was blocked, confirming the receptor’s important role in mediating osteogenic differentiation (*P* < 0.01) (Fig. 8C–F). These data indicate that MT2 plays vital roles in MT-regulated oxidative stress attenuation and osteogenic differentiation.

Finally, we applied shGdf15 to knockdown Gdf15 expression in BMSC to further validate the function of GDF15 in MT-regulated ferroptosis attenuation and osteogenic differentiation (Additional file 1: Figure S2A, B; full-length blots are presented in Additional file 2: Figure S12, S13). After knockdown of Gdf15 in BMSC, 100 µM MP caused more severe cell death (*P* < 0.01), while 100 µM MT did not significantly rescue MP-induced cell death (*P* = 0.1148), despite there was a therapeutic trend (Fig. 8A, B). In addition, although there was no significant difference between the shC and shGdf15 groups (*P* = 0.3047), it appeared that basal cell death levels were increased after knockdown of GDF15 (Fig. 8A, B). This trend became more significant in mitochondrial potential (*P* < 0.05), lipid peroxidation (*P* < 0.05) and ROS scavenging gene Slc7a11 (*P* < 0.05) (Fig. 8C-F), suggesting that GDF15 might play an important role in BMSC by maintaining basal cell activity levels. In addition, the basic osteogenic differentiation ability was also impaired, as detected by ALP (*P* = 0.0924) and ARS (*P* < 0.05), further confirming the importance of GDF15 in cell function (Fig. 8G–J). GDF15 also plays an important role in the therapeutic effects of MT. After knockdown of GDF15, MT treatment failed to restore BMSC mitochondrial potential and lipid peroxidation to basal levels (*P* < 0.01) (Fig. 8C, D). ALP and ARS staining also confirmed that GDF15 knockdown significantly affected the osteogenesis-inducing function of MT compared with MP treatment (ALP: *P* = 0.1760, ARS: *P* = 0.0452) (Fig. 8G–J). However, despite GDF15 knockdown, Slc7a11 expression was also partially induced by MT, and ARS staining showed that MT also promoted a portion of osteogenic differentiation function (Fig. 8E, I, J). This suggests that although GDF15 plays an important role in the therapeutic mechanism of MT, part of the MT function works through other pathways, possibly direct interactions of MT with other proteins.

## Discussion

Patients with ONFH often exhibit symptoms such as hip pain in the early stages; limited mobility, limping, or becoming bedridden can occur in the late stages, leading to a decreased quality of life [43]. However, there is no effective treatment available for ONFH, especially GC-induced cases. In the current study, we found that ferroptosis in BMSCs is closely related to the process of SONFH and showed that MT could attenuate GC-induced bone loss in a rat SONFH model by suppressing ferroptosis. Mechanistically, high-dose GC inhibited the expression of GDF15, which has the ability to regulate the expression of GPX4 and SLC7A11. However, since high-dose GC directly causes ROS production and lipid peroxidation, a decrease in GPX4 and SLC7A11 can exacerbate the equilibrium of redox systems and eventually cause ferroptosis. Fortunately, MT increases the expression of GDF15 and maintains the balance of the redox system in BMSCs. Additionally, MT also presented a good therapeutic effect in a rat SONFH model. Therefore, supplementation with exogenous MT may be a promising method for the treatment of SONFH.

MT is an indoleamine secreted from the mammalian pineal gland that is involved in antiapoptotic, antitumor, and antioxidant processes [44–46]. In recent years, several studies have reported a correlation between MT and bone metabolism. Maria et al. demonstrated that MT induces human mesenchymal stem cell osteoblast differentiation and mineralization [47]. In the present study, MT treatment effectively lowered the SONFH group rats. The micro-CT results showed that MT increased the subchondral bone mass of the femoral heads in GC-induced ONFH rats. Zhou et al. reported that a low dose (10 µM) of MT resembles the approximate physiological concentration and promotes BMSC osteogenic differentiation [33]. Furthermore, Xiao and colleagues demonstrated that osteoporosis was alleviated by continuous release of MT from a composite adhesive hydrogel system and proposed that it may improve implant osseointegration and increase bone mass [48]. These results are consistent with our findings, which indicated that MT attenuated SONFH in rats by increasing bone quality or bone formation, suggesting that it might be a potential treatment strategy for SONFH.

The relative lack of ROS scavengers may be an important cause of disease during high-dose GC-induced ONFH development. Multiple studies have shown that oxygen radicals are involved in disease pathogenesis [49, 50]. Ichiseki et al. showed that SONFH was aggravated by a brief period of oxidative stress [51]. We also found that high doses of MP increased ROS levels in BMSC. Intravenous injection of substances with antioxidative effects, such as vitamin E, coenzyme Q10, proanthocyanidin, and pravastatin, could reduce the incidence of SONFH and inhibit its development in animal models [52, 53]. In an in vitro study, we found that the expression of Gpx4, Sod2 and Hmox1 was upregulated after MT administration in response to MP-induced damage, suggesting that MT supplementation reduced ROS by regulating the antioxidant system. PI staining further confirmed the protective effect of MT; that is, MP-induced cell death could be significantly reduced by MT treatment. However, we found that 100 μM MT treatment could not reduce ROS and PI staining to basal levels. Therefore, we speculated that other types of cell death might be involved in MP-induced injury. According to previous studies, we conclude that a high dose of glucocorticoid can cause multiple types of cell death, including apoptosis and autophagy, and even possibly cause necroptosis and cuproptosis, which are recently proposed cell deaths [54, 55]. Therefore, we speculated that different concentrations of MP would lead to different types of cell death and be dominated by one type, but also accompanied by other types of cell death. In the current study, MT effectively alleviated MP-induced cell death in a dose-dependent manner, and it was confirmed by experiments that this protective effect works through ferroptosis. In future studies, it will be necessary to clarify and standardize the different effects of different concentrations of glucocorticoids on BMSC.

In addition, bioinformatic analysis further showed that ferroptosis played a critical role in promoting the progression of SONFH. Ferroptosis is a novel type of iron-dependent cell death and is characterized by decreased ROS scavenging and increased lipid peroxidation [56, 57]. However, there are few studies on the relationship between ferroptosis and steroid-induced necrosis of the femoral head. In this study, we screened some high-throughput sequencing databases. Fortunately, after searching for gene expression profiles with keywords related to SONFH, we screened out datasets that were treated with high-dose MP. In addition, we also obtained a series of datasets about MT intervention. Then, we analyzed DEGs with a *P* value < 0.05 and |log2FC|≥ 1 and carried out Venn analysis to obtain the intersection of DEGs with the ferroptosis gene set. Finally, we confirmed that GDF15 played an important role in MP treatments and MT treatments. Western blot analyses also verified that GC could inhibit GDF15 expression in BMSC, and this effect was rescued by MT treatments.

MT1 and MT2 receptors are G protein-coupled receptors with seven transmembrane structures [58]. The MT1 receptor is mainly involved in the regulation of reproductive activity and has little impact on bone metabolism [59]. MT2 is the main receptor subtype involved in regulating bone metabolism via MT treatment and MT2 gene polymorphisms [60]; it is widely distributed in the bone, brain, liver, and retina [61]. The osteogenic effect of MT is diminished when MT2 is knocked down [33]. Furthermore, MT supplementation promotes osteogenesis by activating MT2 to upregulate the gene expression of ALP, bone morphogenetic proteins 2 and 6, osteocalcin, and osteoprotegerin [62]. Consistent with these studies, when luzindole, the MT2 receptor antagonist, was applied with MT, the beneficial effects of MT disappeared in MP-treated BMSC. Anti-ferroptosis proteins were inhibited again, and ROS levels were upregulated in the MT and luzindole treatment groups. Therefore, we propose that the MT2 receptor participates in the process of MT treatment for SONFH. The application of the MT2 receptor antagonist luzindole also validated that GDF15 expression was regulated by MT.

However, there are still many unconfirmed parts to be explored. Although MT treatment increased GDF15 expression and inhibition of the MT2 receptor could reduce GDF15 expression again, the mechanism of increases in GDF15 expression needs more investigation to clarify.

## Conclusion

Our results demonstrate that MT alleviated SONFH in a rat model. Mechanistically, MT attenuated GC-induced ROS generation and promoted osteogenic differentiation through MT2-mediated inhibition of ferroptosis. MT might be a suitable candidate for application in the prevention and treatment of SONFH.

## Supplementary Information

**Additional file 1**. **Figure S1**: **A** OCN expression in BMSC was detected with immunofluorescence staining.**B** Mean fluorescence intensity of OCN. **C** ALP staining.**D** Quantitative analysis of ALP activity. **E** ARS staining.**F** Quantitative analysis of ARS.**Figure S2**: **A**, **B** qPCR and western blot analysis of GDF15 in BMSCs after knockdown of GDF15 with shGdf15.

**Additional file 2**.** Figures S3–S13**: This file includes uncropped full-length blots used in the article.

## Data Availability

All data generated and/or analyzed during this study are available from the corresponding author upon reasonable request. The RNA-seq data analyzed in this article has been deposited in NCBI’s Gene Expression Omnibus (GEO) and are accessible through GEO Series accession number GSE183359, GSE190135 and GSE112101.

## Abbreviations

ROS: Reactive oxygen species
GC: Glucocorticoid
MP: Methylprednisolone sodium succinate
BMSC: Bone marrow mesenchymal stem cell
SONFH: Steroid-induced osteonecrosis of the femoral head
MT: Melatonin
GDF15: Growth differentiation factor 15
GPX4: Glutathione peroxidase 4
GSH: Glutathione
SLC7A11: Solute carrier family 7 member 11
Runx2: Runt-related transcription factor 2
OCN: Osteocalcin
ALP: Alkaline phosphatase
ARS: Alizarin red S
SOD: Superoxide dismutase
Hmox-1: Heme oxygenase-1
NOX4: NADPH oxidase 4
