# Supplementary material for: Exogenous melatonin ameliorates steroid-induced osteonecrosis of the femoral head by modulating ferroptosis through GDF15-mediated signaling
Source: Stem Cell Res Ther. 2023 Jul 3;14:171. doi: 10.1186/s13287-023-03371-y (PMC10318673; doi:10.1186/s13287-023-03371-y)
Supplement: Supplementary file 1 — Additional file 1. Figure S1: A OCN expression in BMSC was detected with immunofluorescence staining.B Mean fluorescence intensity of OCN. C ALP staining.D Quantitative analysis of ALP activity. E ARS staining.F Quantitative analysis of ARS.Figure S2: A, B qPCR and western blot analysis of GDF15 in BMSCs after knockdown of GDF15 with shGdf15. [file 13287_2023_3371_MOESM1_ESM.docx]

**Supplementary Materials**

**Exogenous Melatonin Ameliorates Steroid-Induced Osteonecrosis of The Femoral Head by Modulating Ferroptosis through GDF15-Mediated Signaling**

**Wenming Li^a, 1^, Wenhao Li^a, 1^, Wei Zhang^a, 1^, Hongzhi Wang^b^, Lei Yu^a^, Peng Yang^a^, Yi Qin^a^, Minfeng Gan^a^, Xing Yang^c^, Lixin Huang^a^, Yuefeng Hao^c,^ *, Dechun Geng^a,^ ***

**Supplementary Figure S1**

**
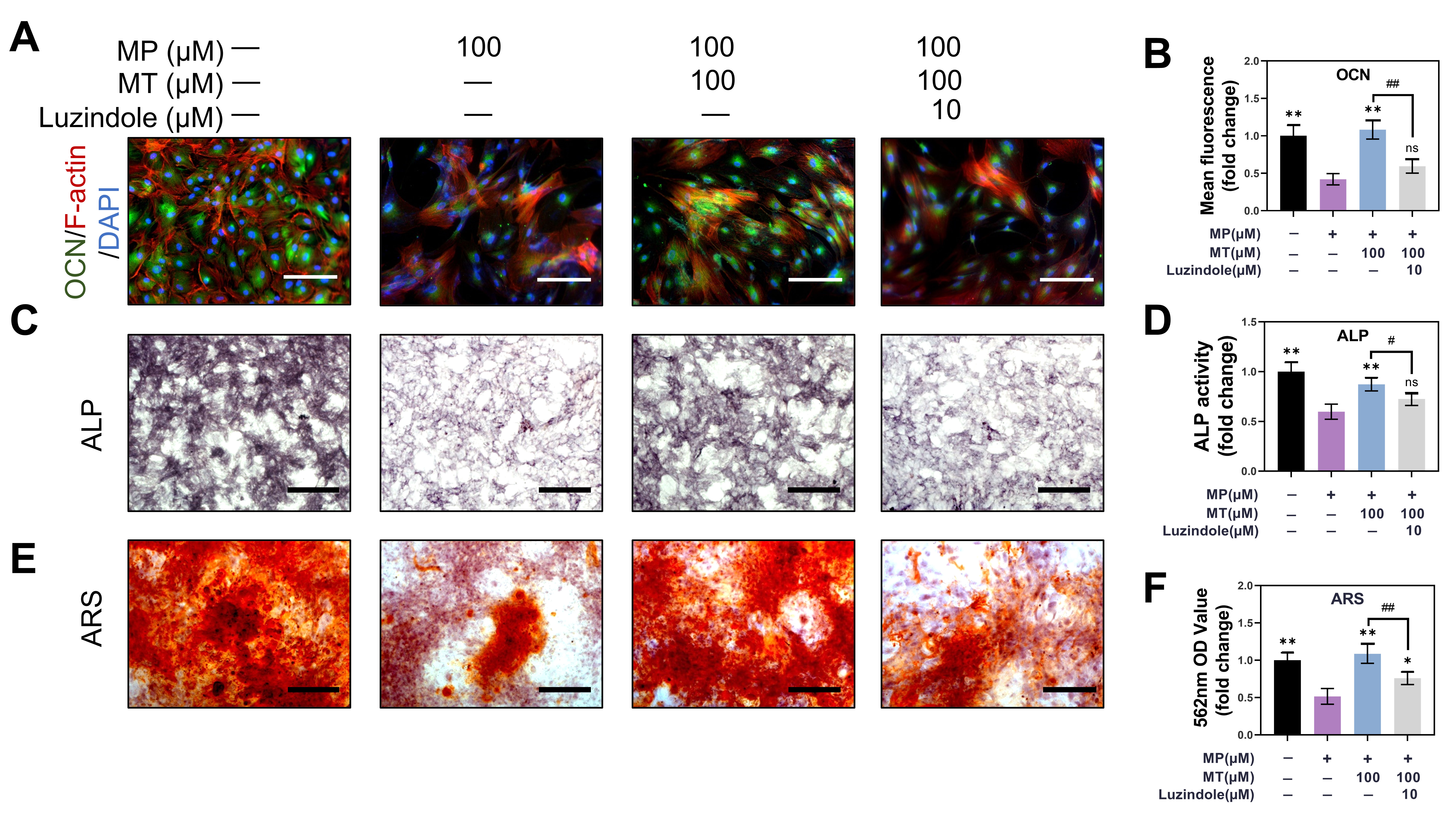
**

**Figure S1** (A) OCN expression in BMSC was detected with immunofluorescence staining. (OCN stained with Alexa Fluor® 488 and F-actin stained with rhodamine. Scale bar: 50 μm.) (B) Mean fluorescence intensity of OCN. (C) ALP staining. (Scale bar: 100 μm) (D) Quantitative analysis of ALP activity. (E) ARS staining. (Scale bar: 100 μm) (F) Quantitative analysis of ARS. (*p < 0.05 and **p < 0.01 compared with the 100 μM MP treatment only group. #p < 0.05 and ##p < 0.01 are comparisons between the 100 μM MT and 10 μM luzindole treatment groups.)

**Supplementary Figure S2**

**
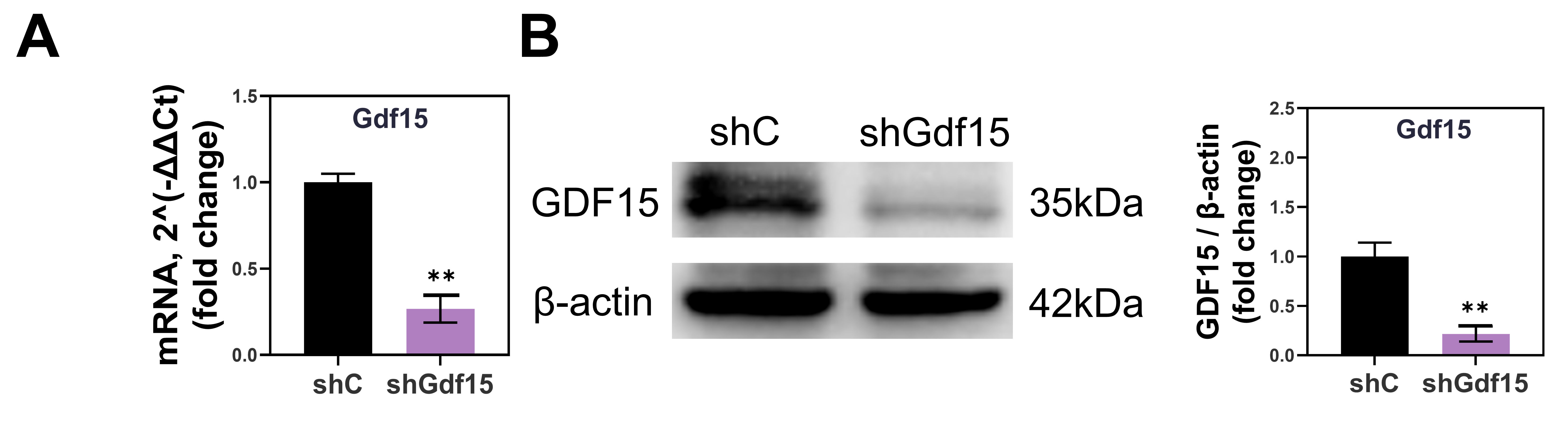
**

**Figure S2** (A), (B) qPCR and western blot analysis of GDF15 in BMSCs after knockdown of GDF15 with shGdf15. (**p < 0.01 compared with the shC group. Full-length blots are presented in supplementary **Figure S12-S13**.)
