# Supplementary material for: Exogenous melatonin ameliorates steroid-induced osteonecrosis of the femoral head by modulating ferroptosis through GDF15-mediated signaling
Source: Stem Cell Res Ther. 2023 Jul 3;14:171. doi: 10.1186/s13287-023-03371-y (PMC10318673; doi:10.1186/s13287-023-03371-y)
Supplement: Supplementary file 2 — Additional file 2. Figures S3–S13: This file includes uncropped full-length blots used in the article. [file 13287_2023_3371_MOESM2_ESM.docx]

**Supplementary Materials**

**Exogenous Melatonin Ameliorates Steroid-Induced Osteonecrosis of The Femoral Head by Modulating Ferroptosis through GDF15-Mediated Signaling**

**Wenming Li^a, 1^, Wenhao Li^a, 1^, Wei Zhang^a, 1^, Hongzhi Wang^b^, Lei Yu^a^, Peng Yang^a^, Yi Qin^a^, Minfeng Gan^a^, Xing Yang^c^, Lixin Huang^a^, Yuefeng Hao^c,^ *, Dechun Geng^a,^ ***

This file includes uncropped full-length blots used in the article. For each original uncropped full-length blots, we provide 2 images: one was taken by chemiluminescence, and the other one was taken in the bright field.

**Supplementary Figure S3**

**Chemiluminescence Bright field**

**
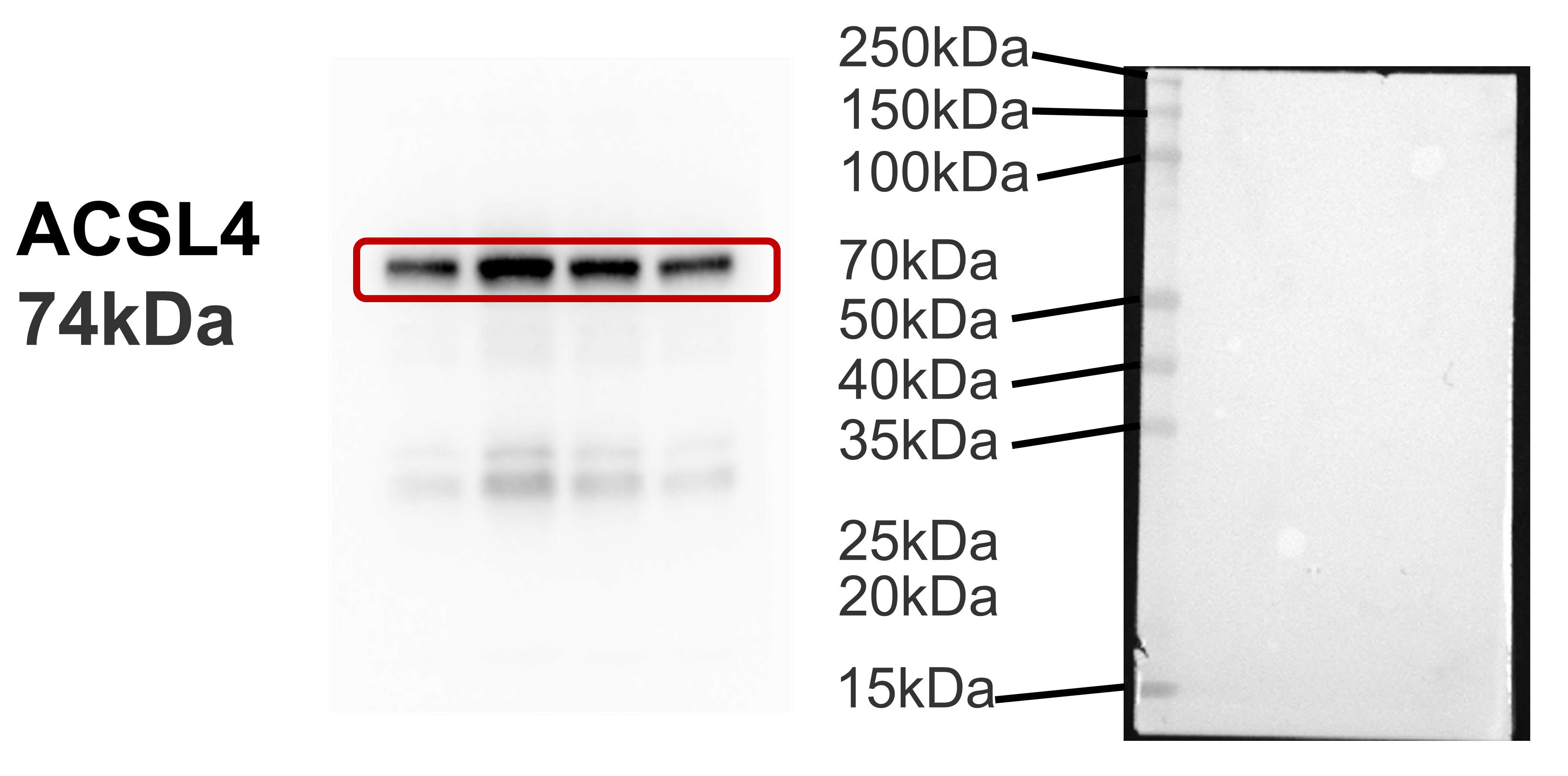
**

**Figure S3** Full-length blots of ACSL4 in Figure 5H.

**Supplementary Figure S4**

**
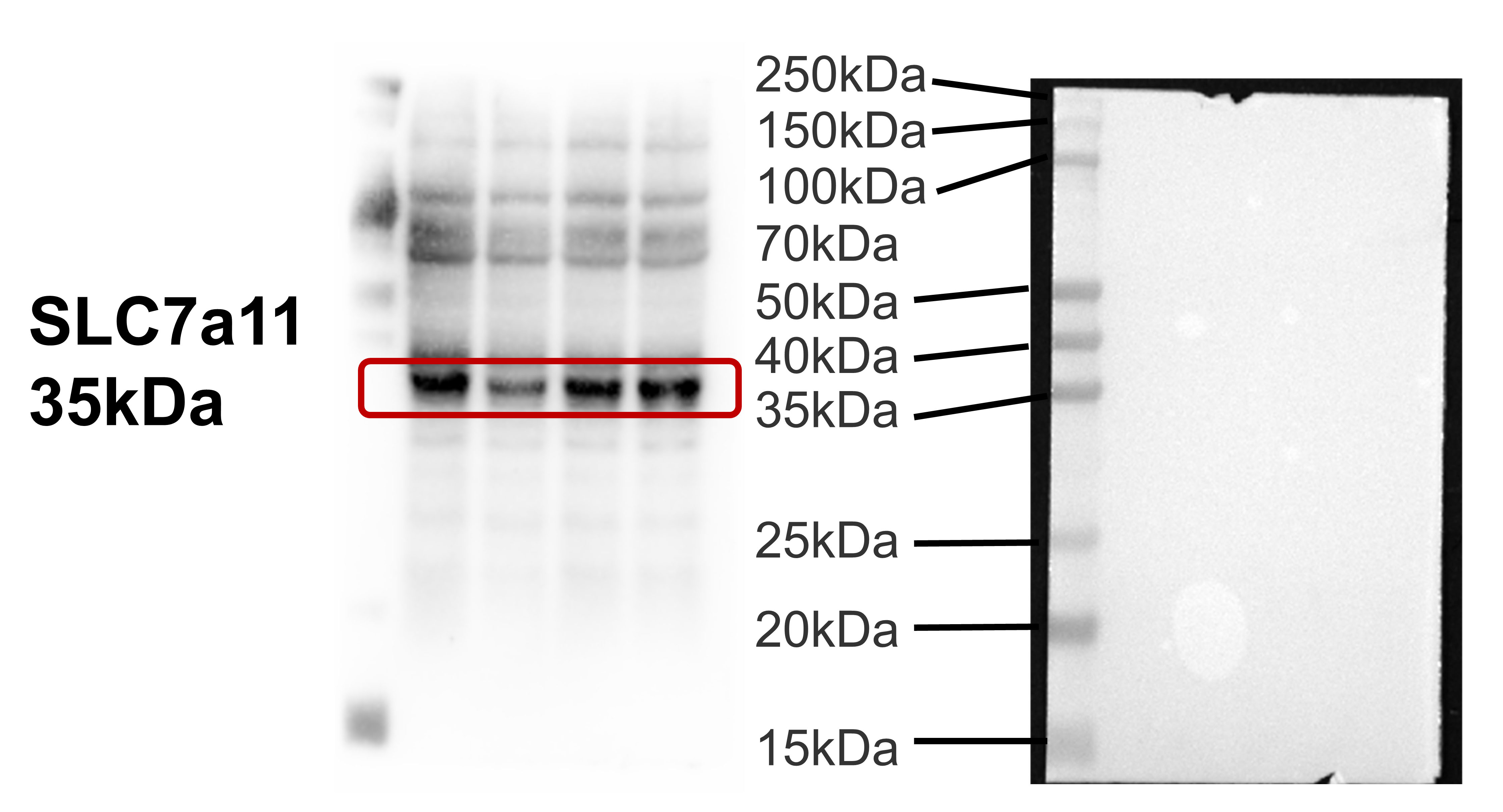
**

**Figure S4** Full-length blots of SLC7a11 in Figure 5H.

**Supplementary Figure S5**

**
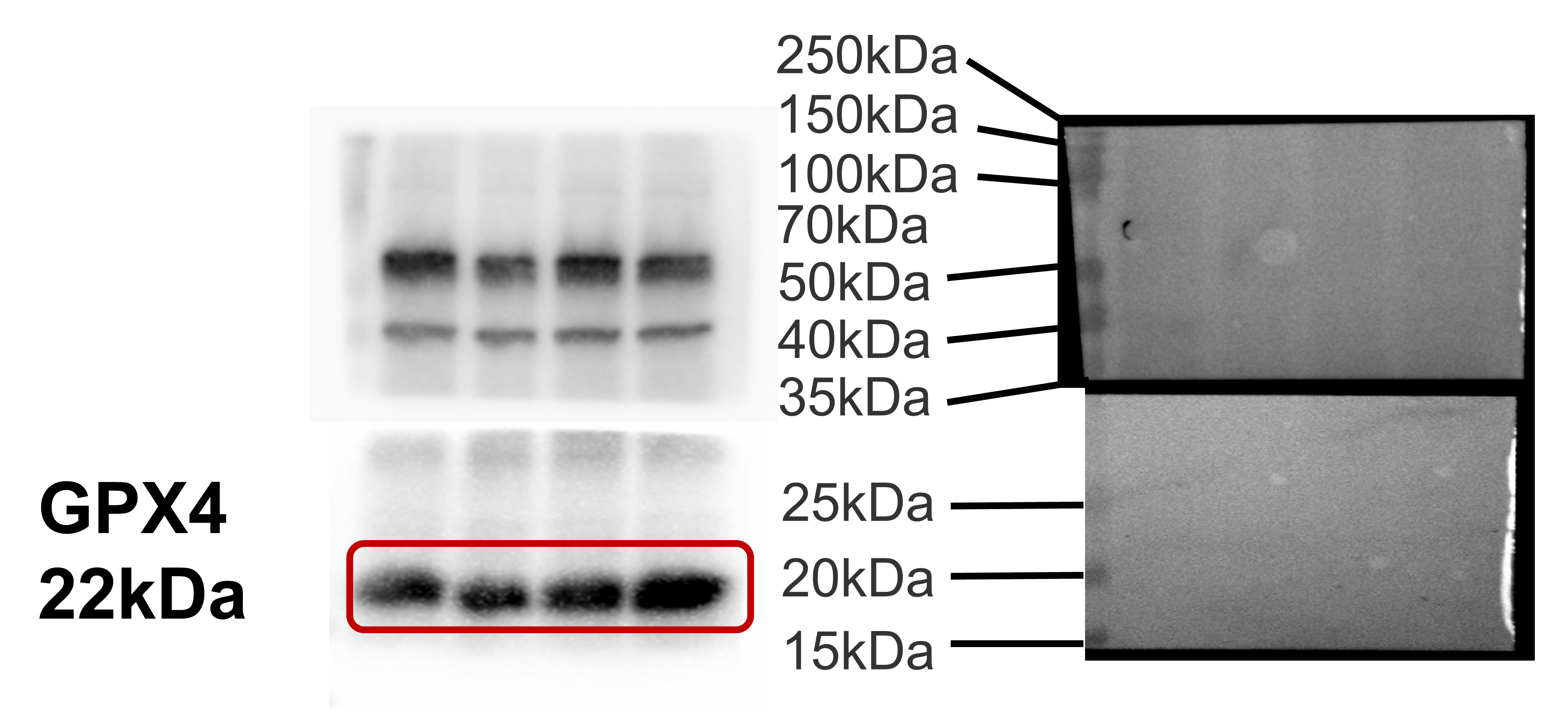
**

**Figure S5** Full-length blots of GPX4 in Figure 5H.

**Supplementary Figure S6**

**
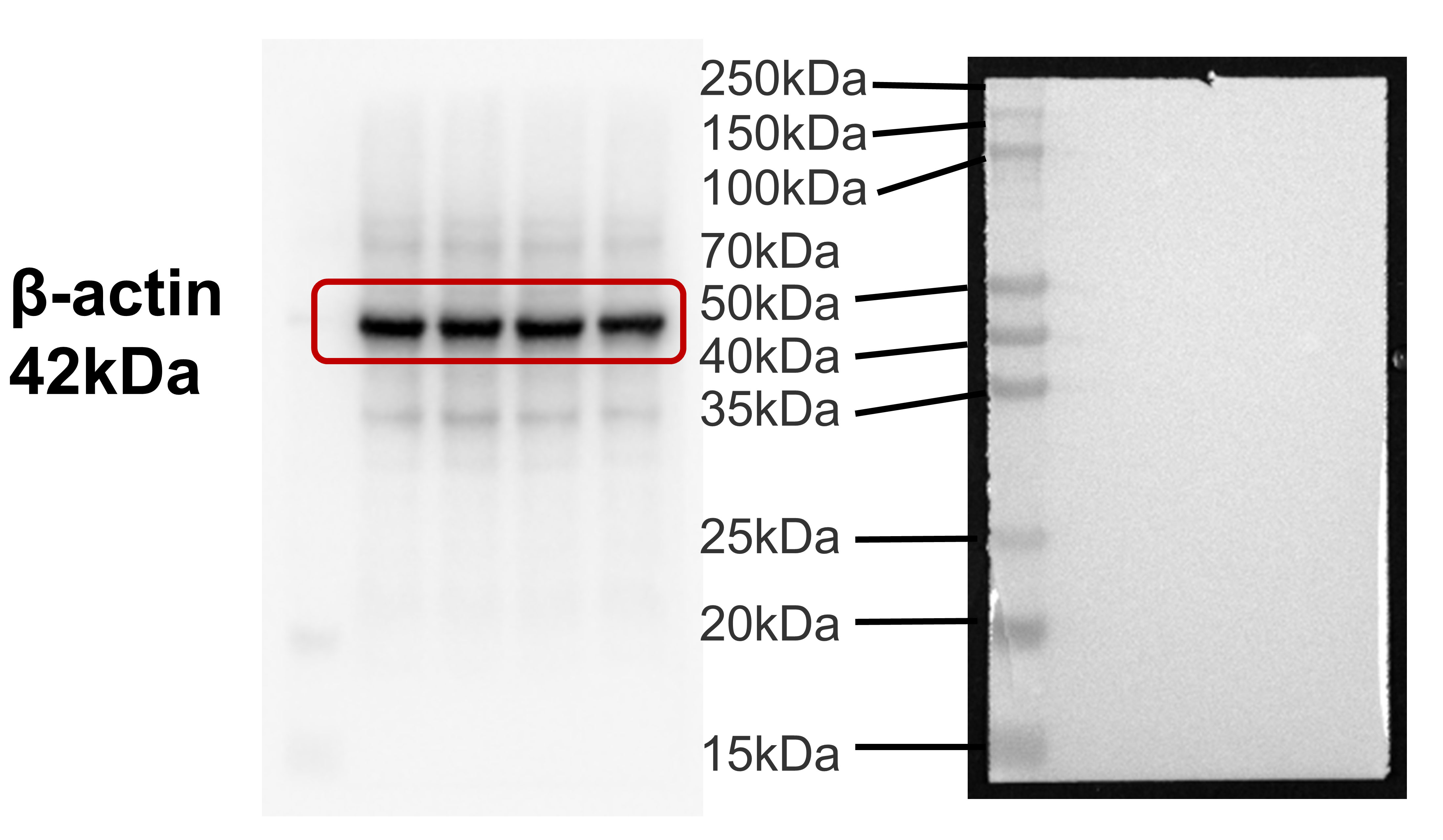
**

**Figure S6** Full-length blots of β-actin in Figure 5H.

**Supplementary Figure S7**

**
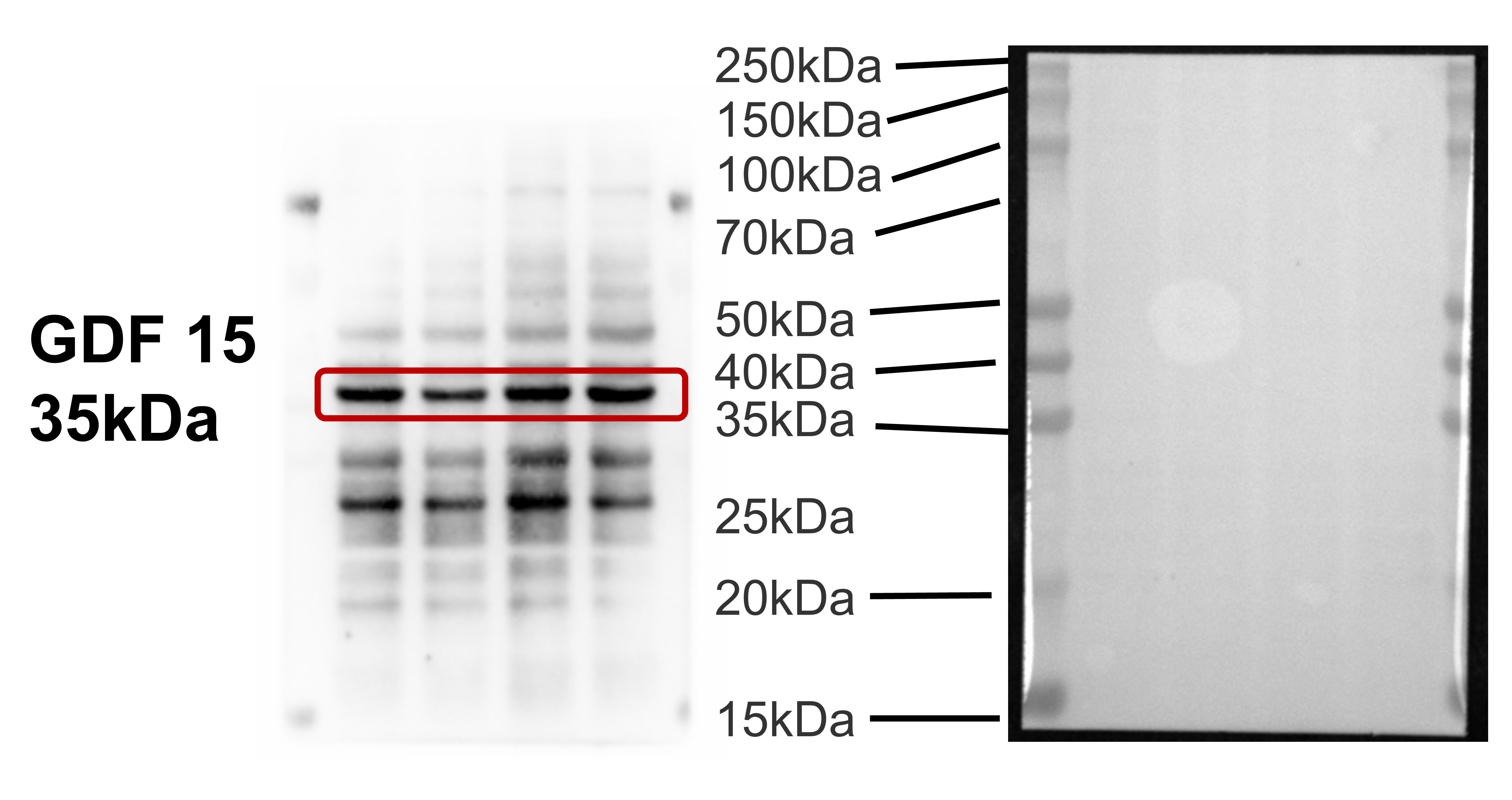
**

**Figure S7** Full-length blots of GDF15 in Figure 6D.

**Supplementary Figure S8**

**
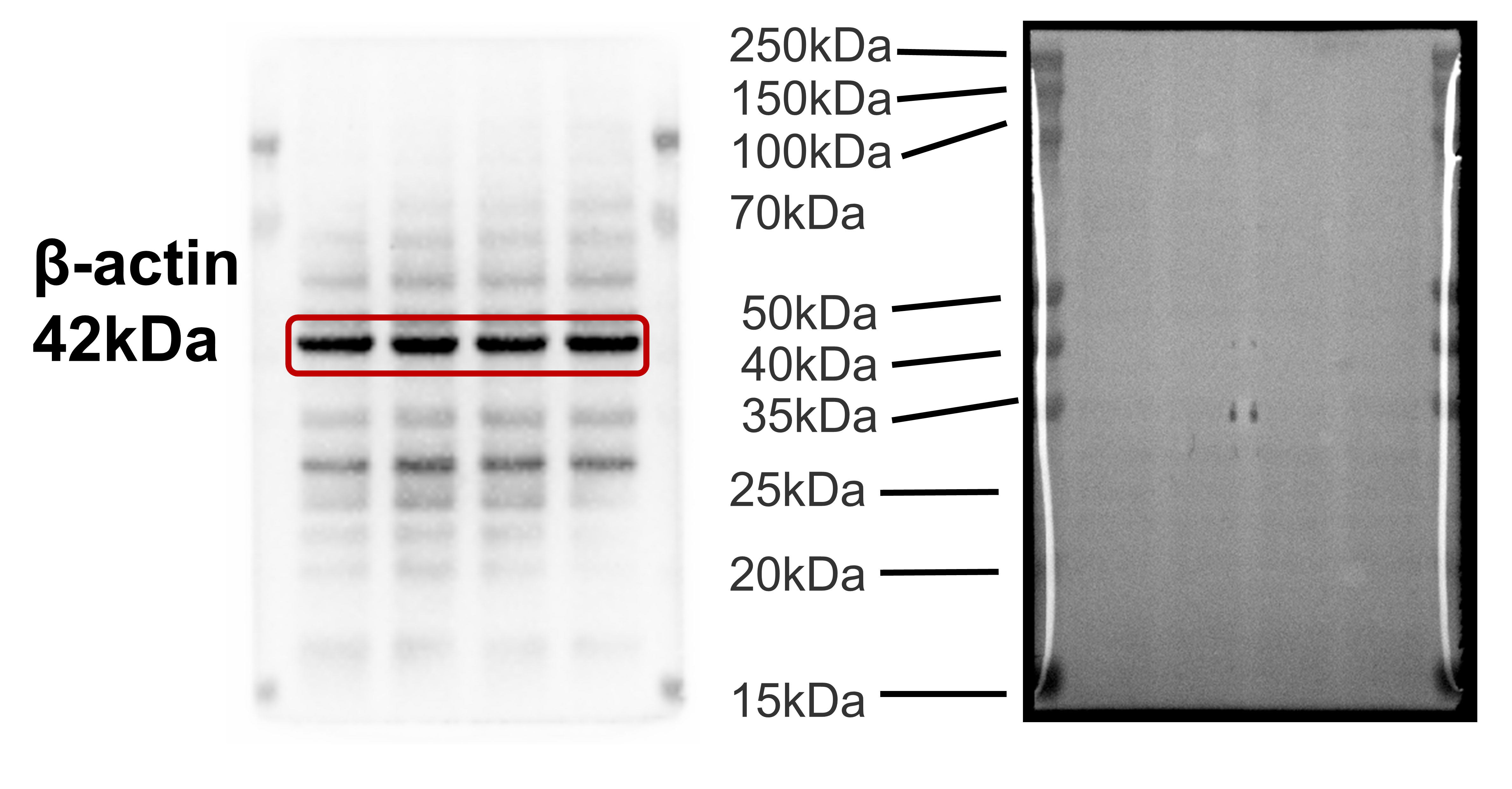
**

**Figure S8** Full-length blots of β-actin in Figure 6D.

**Supplementary Figure S9**

**
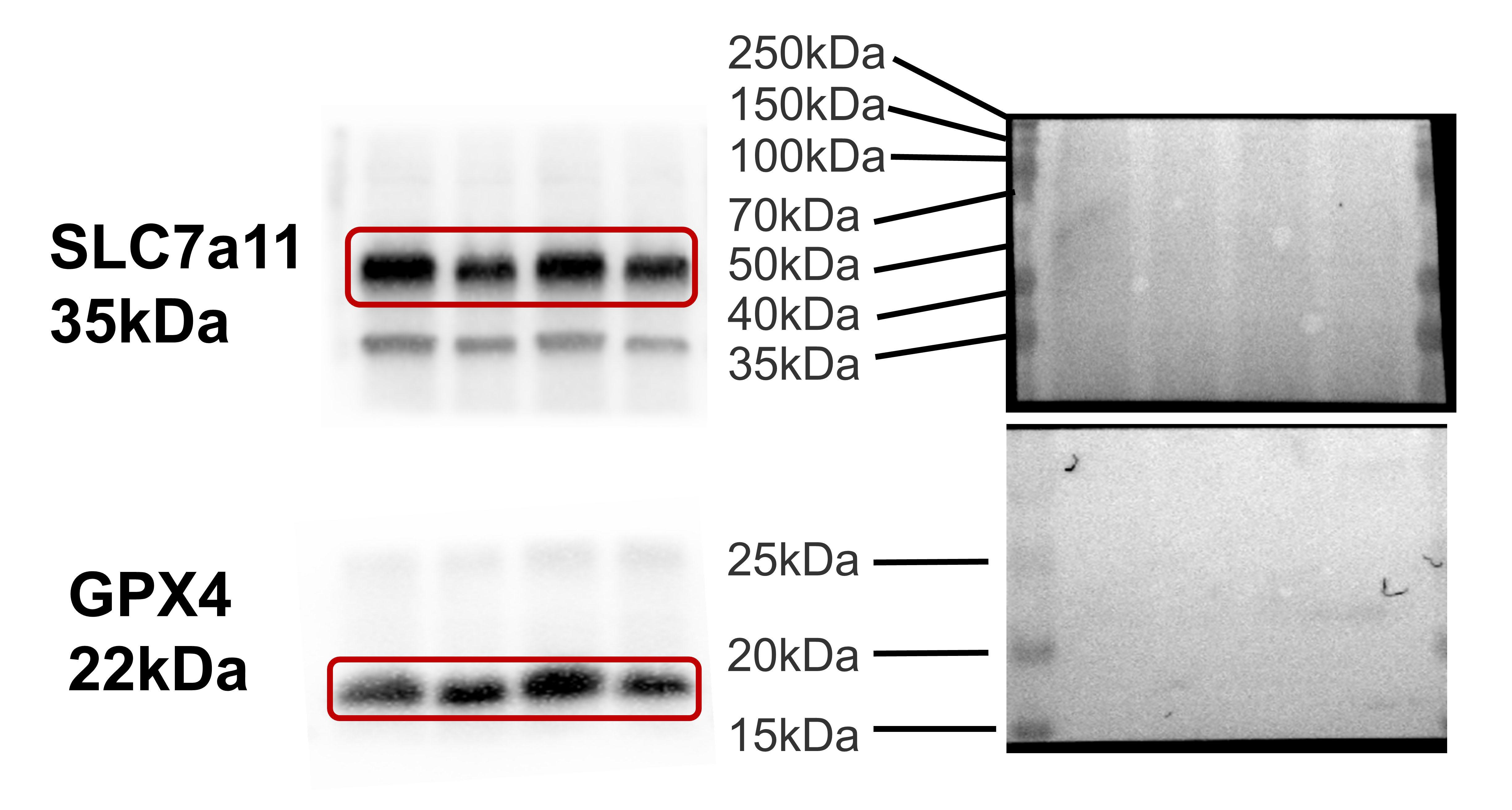
**

**Figure S9** Full-length blots of SLC7a11 and GPX4 in Figure 7E.

**Supplementary Figure S10**

**
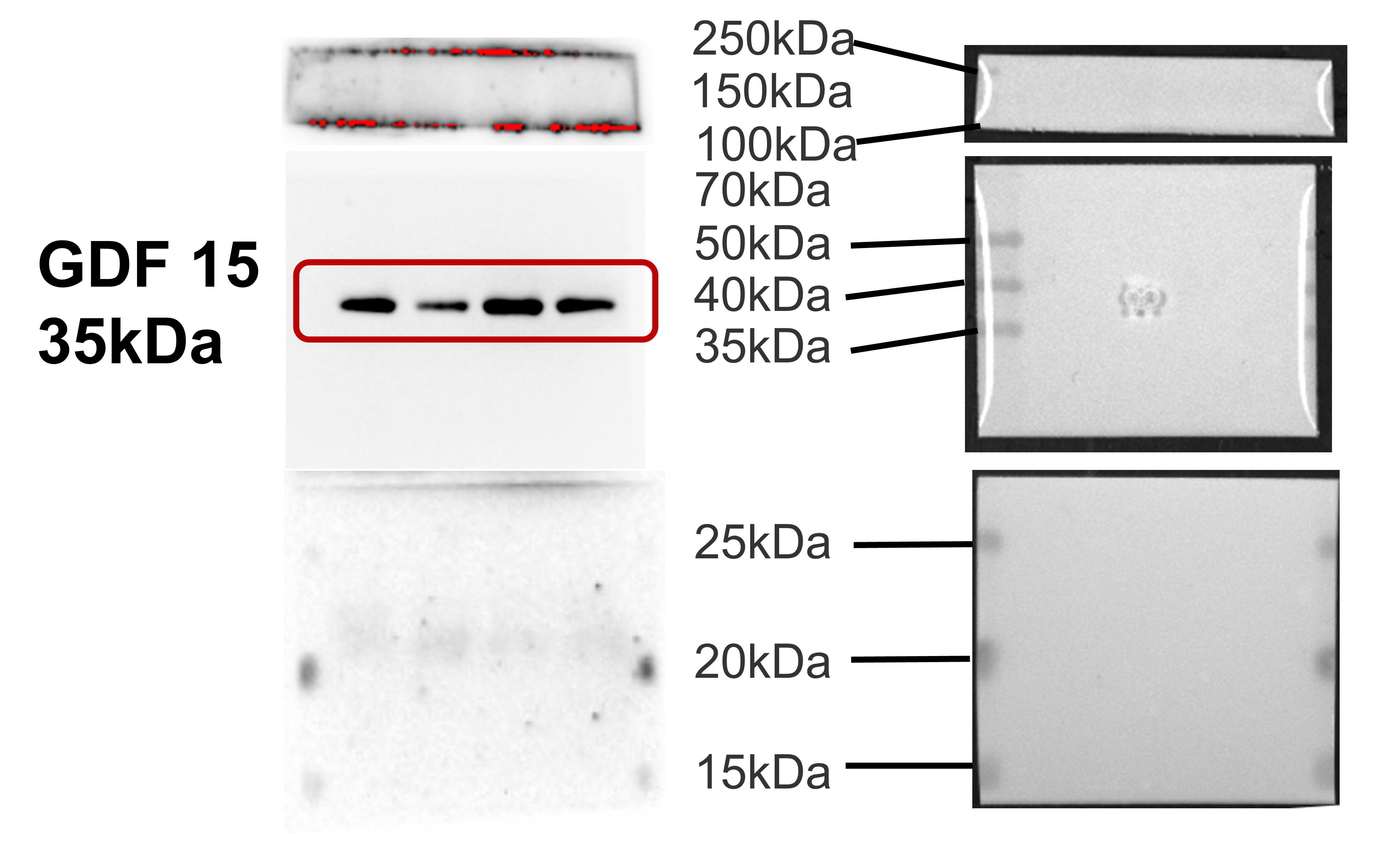
**

**Figure S10** Full-length blots of GDF15 in Figure 7E.

**Supplementary Figure S11**

**
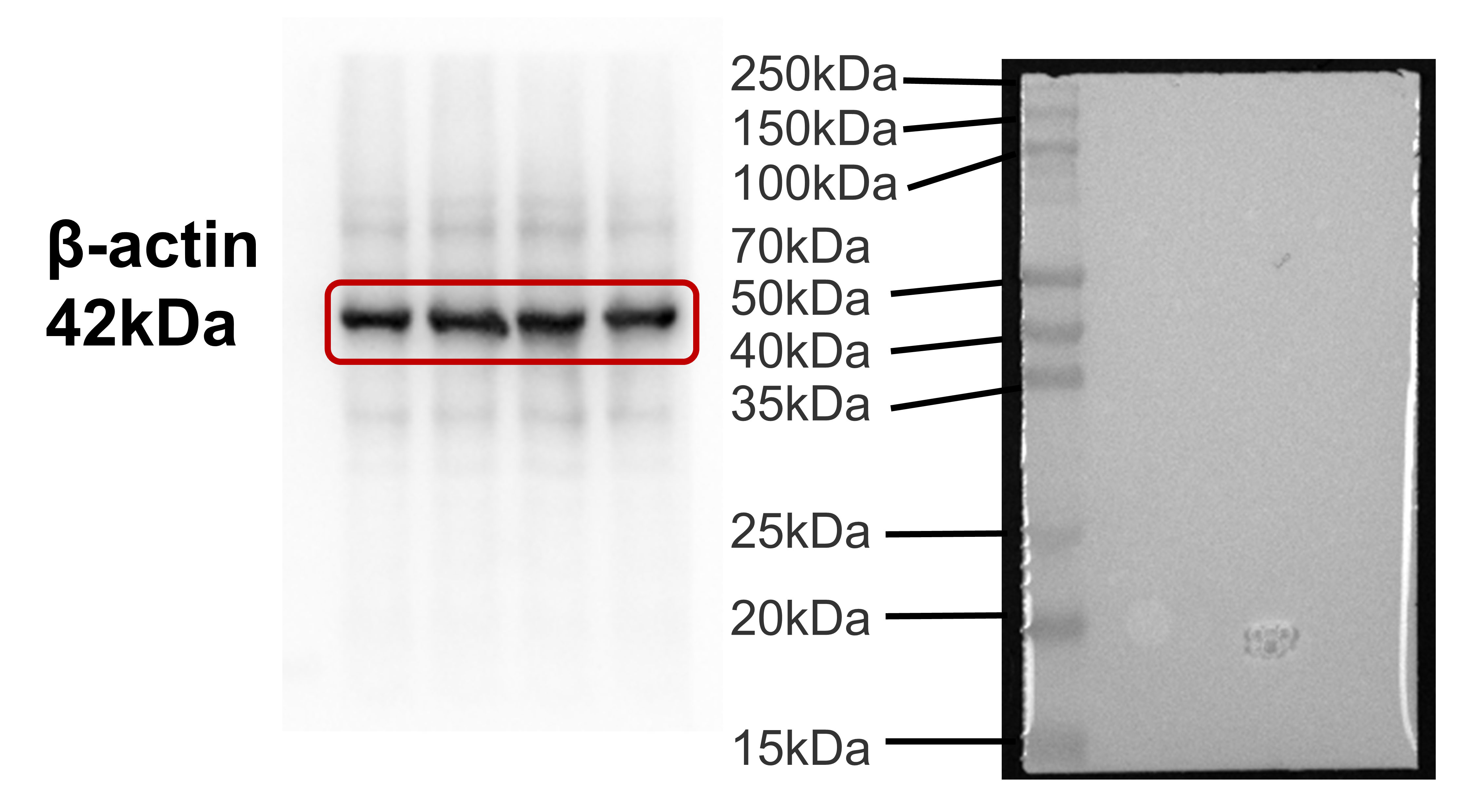
**

**Figure S11** Full-length blots of β-actin in Figure 7E.

**Supplementary figure S12**

**
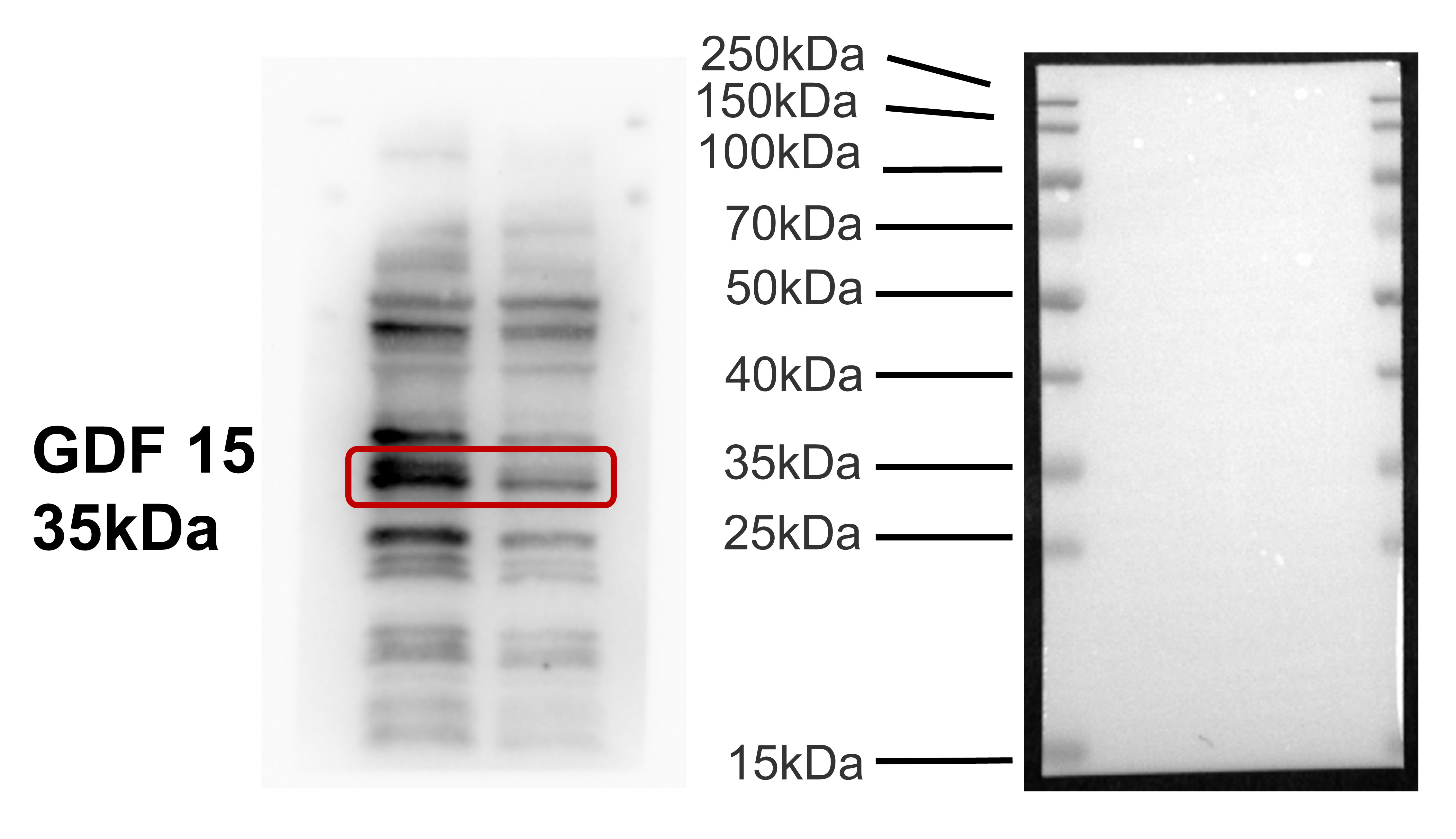
**

**Figure S12** Full-length blots of GDF15 in Figure S12B.

**Supplementary figure S13**


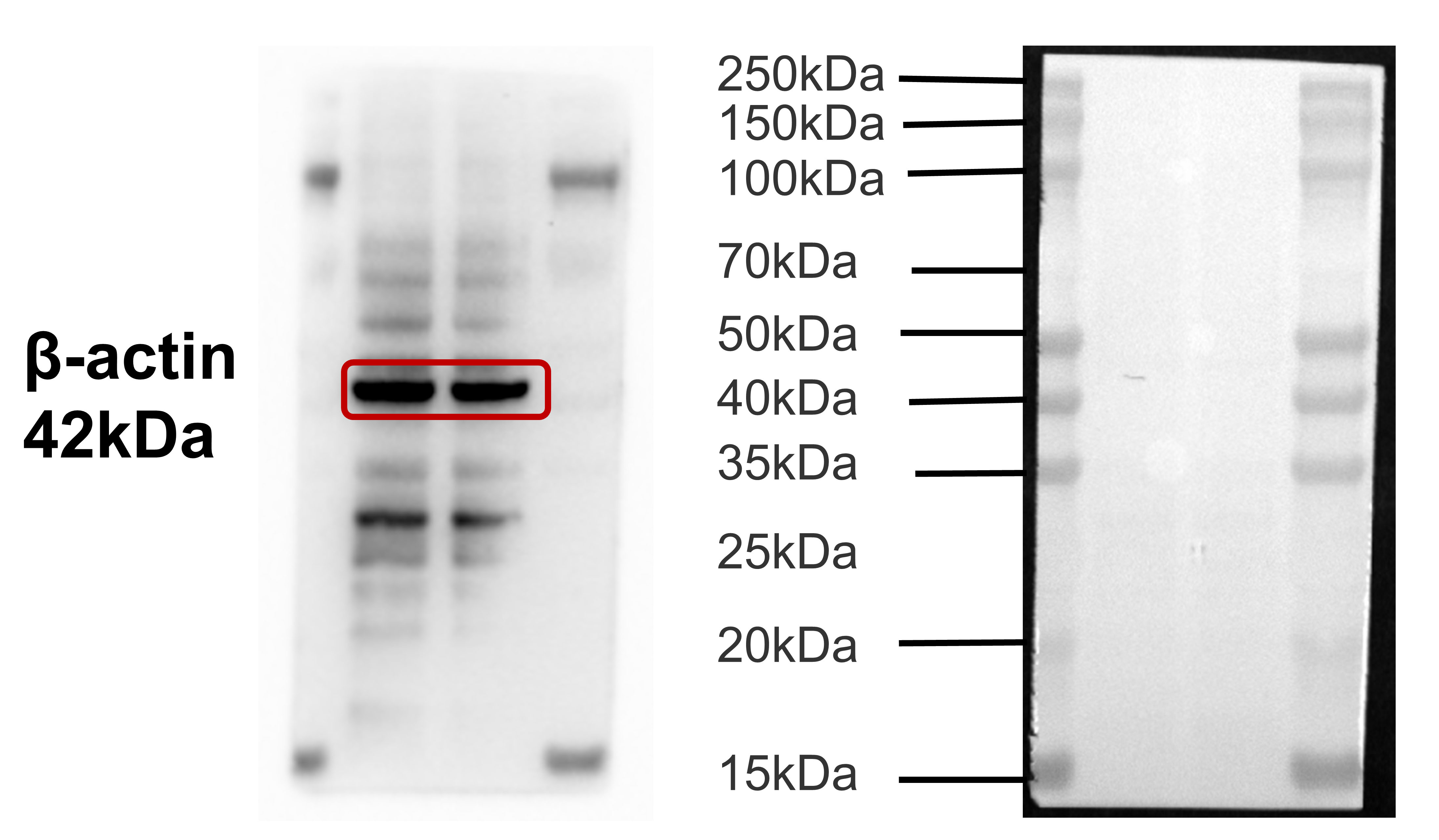


**Figure S13** Full-length blots of β-actin in Figure S12B.
